# Benthic animal-borne sensors and citizen science combine to validate ocean modelling

**DOI:** 10.1038/s41598-022-20254-z

**Published:** 2022-10-05

**Authors:** Edward Lavender, Dmitry Aleynik, Jane Dodd, Janine Illian, Mark James, Sophie Smout, James Thorburn

**Affiliations:** 1 Centre for Research into Ecological and Environmental Modelling, University of St Andrews, St Andrews, UK; 2 Scottish Oceans Institute, University of St Andrews, St Andrews, UK; 3 Scottish Association for Marine Science, Oban, UK; 4 NatureScot, Oban, UK; 5 School of Mathematics and Statistics, University of Glasgow, Glasgow, UK; 6 School of Biological Sciences, Queen’s University Belfast, Belfast, UK

**Keywords:** Marine biology, Physical oceanography

## Abstract

Developments in animal electronic tagging and tracking have transformed the field of movement ecology, but interest is also growing in the contributions of tagged animals to oceanography. Animal-borne sensors can address data gaps, improve ocean model skill and support model validation, but previous studies in this area have focused almost exclusively on satellite-telemetered seabirds and seals. Here, for the first time, we develop the use of benthic species as animal oceanographers by combining archival (depth and temperature) data from animal-borne tags, passive acoustic telemetry and citizen-science mark-recapture records from 2016–17 for the Critically Endangered flapper skate (*Dipturus intermedius*) in Scotland. By comparing temperature observations to predictions from the West Scotland Coastal Ocean Modelling System, we quantify model skill and empirically validate an independent model update. The results from bottom-temperature and temperature-depth profile validation (5,324 observations) fill a key data gap in Scotland. For predictions in 2016, we identified a consistent warm bias (mean = 0.53 °C) but a subsequent model update reduced bias by an estimated 109% and improved model skill. This study uniquely demonstrates the use of benthic animal-borne sensors and citizen-science data for ocean model validation, broadening the range of animal oceanographers in aquatic environments.

## Introduction

In the last three decades, electronic tagging and tracking technologies have been widely applied to study animal movement^1–4^. In aquatic environments, animal-borne sensors have been used to reconstruct fine-scale movements in coastal areas^3^, record-breaking dives^5^ and transoceanic movements of a range of taxa^6,7^. At the same time, animal-borne sensors have become capable of collecting large amounts of oceanographic data and interest is growing in their potential contributions to ocean observing systems and modelling^8–11^.

Recent work has shown how oceanographic data from animal-borne sensors can be used to fill gaps in ocean observing systems^9,10,12^, improve ocean model skill^12,13^ and support data or model validation^14,15^. This is important because in many regions existing ocean observing systems are sparse and the data required for model development and validation are limited^16,17^. For example, studies on seabirds have extracted information on ocean surface currents from drifting individuals^18,19^ and the assimilation of these data into ocean models in some settings has improved the description of ocean processes^13^. Similarly, seabird flight paths have been used to study wind and atmospheric conditions^20–22^, the movements of polar bears (*Ursus maritimus*) have been used to study sea ice dynamics^23^ and dropped (passively drifting) telemetry collars have been used to validate modelled sea ice drift^14,15^. Below the water surface, the use of animal-borne sensors for oceanographic data collection has concentrated on diving mammals, such as elephant seals (*Mirounga sp.*). These animals can be captured and tagged with satellite tags when hauled out on land and subsequently transmit location and oceanographic data packets upon surfacing^11^. Seal-borne conductivity-temperature-depth profiles collected in this way^24,25^ have been assimilated into Antarctic circumpolar ocean circulation models and improved the description of mixed layer properties^12,26^. However, the use of animal-borne sensors in other settings remains underdeveloped, especially near seafloor environments where oceanographic datasets remain particularly limited^16^.

Benthic animals, such as skate (Rajidae), spend their lives near the seabed and are increasingly studied using electronic tagging and tracking^27^. Unlike seals, for benthic (and demersal) species near real-time satellite geolocation is precluded by the absence of a surface phase and alternative tracking technologies are exploited^27^. For example, passive acoustic telemetry couples animal-borne acoustic transmitters with networks of acoustic receivers that detect transmissions that occur within range^2,3,27^. Detections at receivers indicate location and are often associated with sensor (ancillary) data, such as depth or temperature records, that can be retrieved from receivers at periodic intervals. Animal-borne archival tags are also widely deployed to collect depth and temperature records as part of research into vertical movement, behaviour and habitat preferences^2,27^. Archival records can be retrieved from recaptured animals or (in the case of pop-up satellite tags) following tag detachment, via tag recovery or satellite uplink. These technologies have dramatically improved our understanding of the movements of benthic animals^27^, but to date their potential (additional) contributions as sources of benthic oceanographic data for ocean model validation have remained unrealised.

The Finite Volume Coastal Ocean Model (FVCOM) is a widely used primitive-equation, free-surface, hydrostatic model that calculates hydrodynamic conditions in three dimensions across an unstructured, triangular prismatic mesh^28^. Scalar ocean variables, such as temperature, are resolved at prism vertices (nodes), while current-velocity vectors are resolved at prism centroids (elements), for each depth layer from the surface to the seabed. FVCOM is integrated in many regional ocean models, such as the West Scotland Coastal Ocean Modelling System (WeStCOMS)^29,30^. Nested within the North-East Atlantic Regional Ocean Modelling System (NEA-ROMS)^31^, WeStCOMS resolves hydrodynamic conditions across a 40,000 km^2^ area. The boundary forcing is derived from NEA-ROMS and the meteorological forcing is derived from the localised Weather Research and Forecasting (WRF) model^32^. In 2017, the boundary forcing was adjusted to mitigate biases detected in temperature profiles interpolated from the parent model from the surface to the seabed revealed by data from gliders deployed near the model’s south-western boundary. This exercise demonstrated the benefits of in situ data for model validation, but data acquisition from the rest of the model’s domain has remained lacking. In this context, WeStCOMS is an ideal modelling system with which to develop the use of mobile benthic species for ocean model validation.

The flapper skate (*Dipturus intermedius*) is a Critically Endangered benthic elasmobranch^33^ that is found off the west coast of Scotland where data from animal-borne tags have been collated to guide management of a 741 km^2^ Marine Protected Area (MPA) established for its conservation^34–42^. Since the 1970s, a wealth of citizen-science mark-recapture data has been assembled from a recreational catch-and-release sport fishery that principally targets skate from anchored charter vessels over areas of relatively deep (> 100 m) water^34–40^. More recently (2016–17), the flapper skate became the focus of a major electronic tagging and tracking project that deployed passive acoustic telemetry and archival tags^39–41,43^. Collectively, these data provide a novel opportunity to develop the use of benthic species as animal oceanographers and support model validation across a central portion of the WeStCOMS domain.

**Figure 1:**
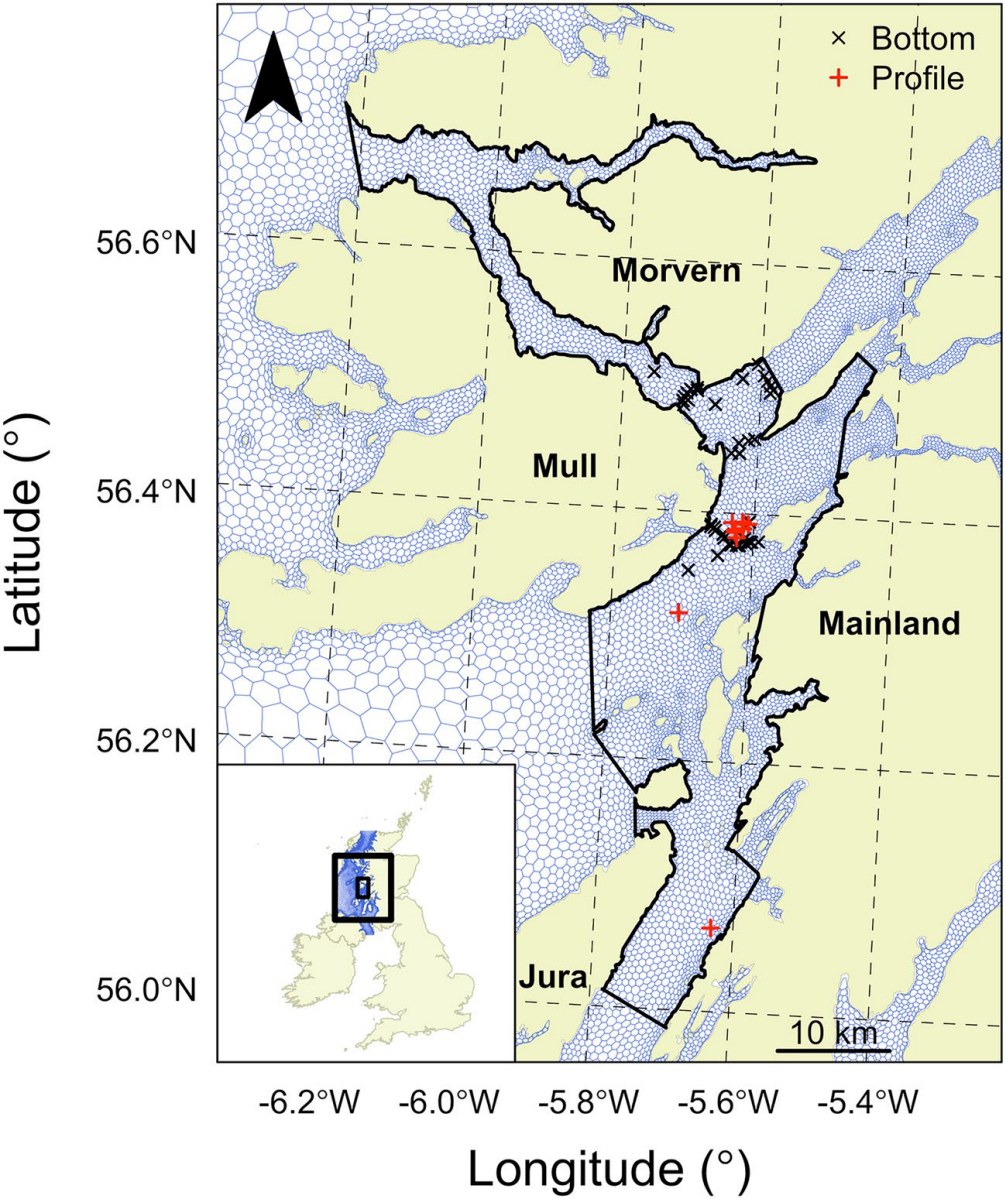
The study site. The inset shows the location of the study site in the British Isles, with the WeStCOMS mesh shown in blue and the study site enclosed within the black rectangles. The main figure shows the study site, including the mesh around nodes (connecting the elements in the native triangular mesh) and the Marine Protected Area (in black). Acoustic receivers that recorded detections associated with bottom-temperature observations recorded by archival tags are marked in black ($n$ = 40). Recreational angling locations associated with temperature-depth profiles are marked in red ($n$ = 8). The coordinate reference system is British National Grid and the north arrow points to grid north. Gridlines mark lines of longitude and latitude. Coastline data were sourced from the Database of Global Administrative Areas^66^. For a higher resolution map, see Supplementary Fig. S1.

**Figure 2:**
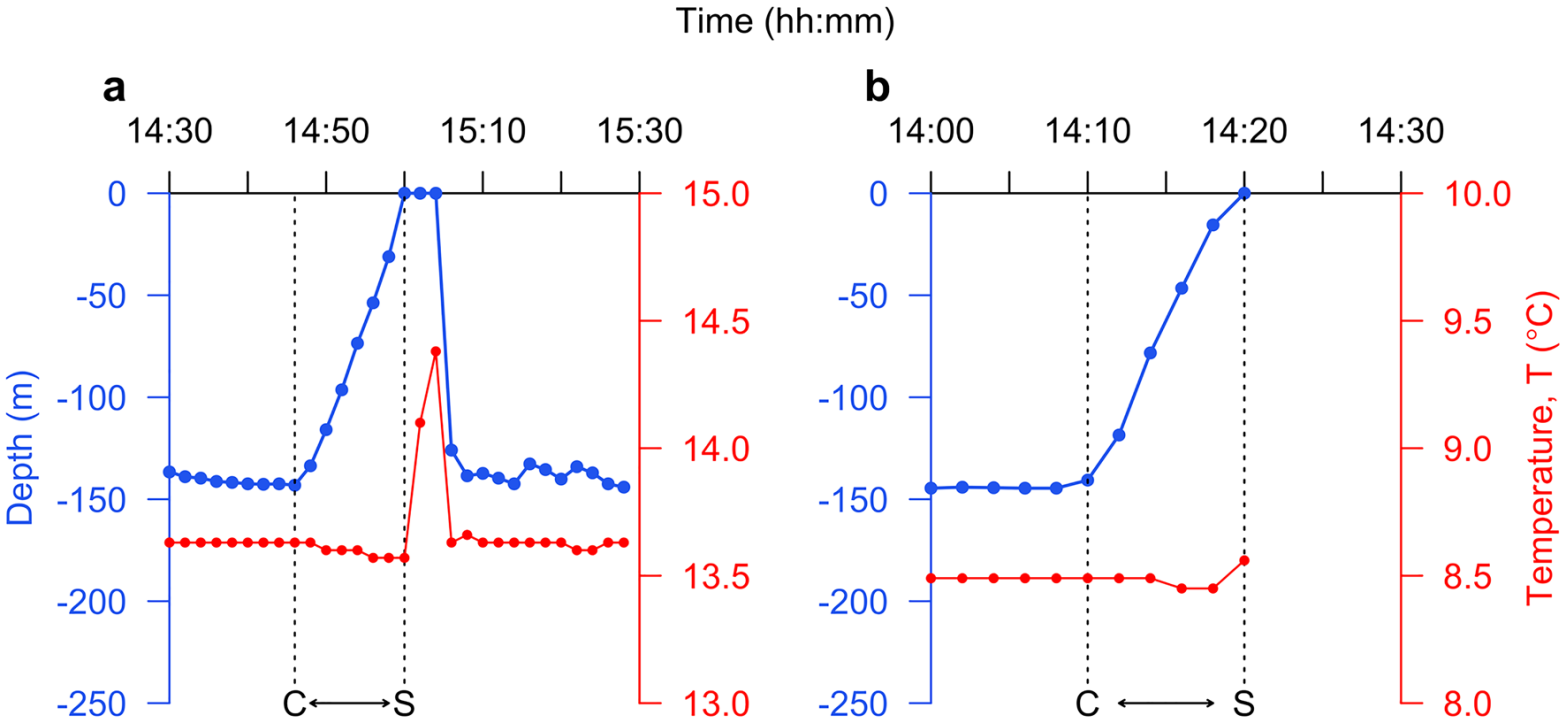
Temperature-depth profiles collected from individual ascents associated with recreational angling events, including (**a**) angling events that occurred during individuals’ time at liberty and (**b**) archival tag retrieval events, in known locations were used to validate modelled temperature-depth profiles**.** Blue lines show depth time series (left axis) and red lines show temperature time series (right axis). Ascents were defined from the time of the last depth observation preceding the capture ascent (labelled C) to the time of the first observation at the surface (labelled S).

The aim of this study is to advance the use of benthic animals in passive acoustic telemetry systems as sources of oceanographic data for ocean model validation. With the flapper skate and WeStCOMS as a case study, we used acoustic detections at receivers and citizen-science mark-recapture records to localise tagged individuals and simultaneous temperature observations measured by animal-borne archival tags to validate modelled temperatures for the seabed (during undisturbed activity) and the water column (during recreational angling events) (Figs. 1–2 and Supplementary Fig. S1). Given the time window of observations (2016–17), we also exploited a unique opportunity to quantify the improvement in model skill resulting from an independent model update during this time.

## Results

### Bottom-temperature validation

**Figure 3:**
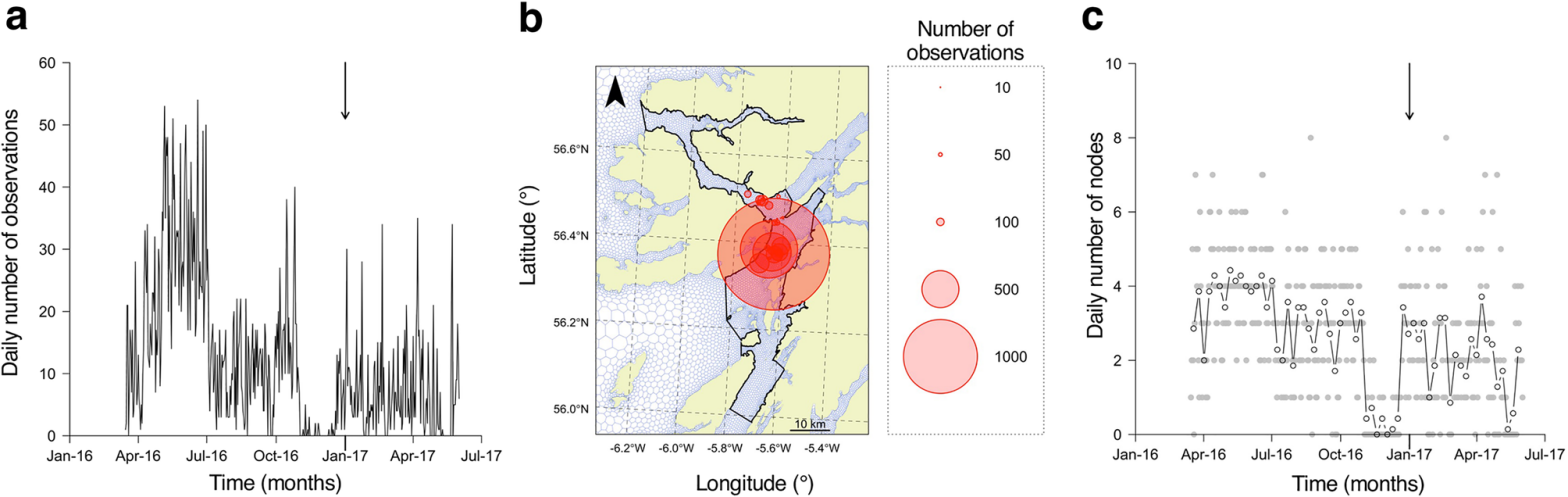
Bottom-temperature validation effort. (**a**) Temporal effort, expressed as the number of observations per day through time. The arrow marks the transition from 2016 to 2017; observations before and after this transition span a critical window when the model was independently upgraded. (**b**) Spatial effort, expressed as the number of observations at each receiver location. Map properties follow Fig. 1. (**c**) Spatiotemporal effort, expressed as the number of nodes with observations per day through time. The filled grey points show the number of nodes for each time step (hour); the open points and the line mark the weekly mean.

For the validation of modelled bottom temperatures, the integration of archival temperature and passive acoustic telemetry data led to a validation dataset comprising 5,260 observations from 14 individuals across all hours of the day and 37 nodes (Supplementary Table S1). The negligible variation in modelled bottom temperatures for these 37 nodes compared to neighbouring nodes (median absolute difference = 0.005 °C) and through time (median absolute hourly difference = 0.010 °C) suggests that the interpolation method used to create the validation dataset was appropriate. Over time, validation effort exceeded one year in duration (from 15^th^ March 2016 to 1^st^ June 2017), although there were 74 days without any observations during this time (Fig. 3a). The number of observations ($n$) was relatively high in spring/summer 2016 and more limited thereafter. Over space, the distribution of validation effort concentrated in the centre of the study site, with 29% of observations at one node (22,420) at a depth of 96 m, a further 10–15% of observations at each of two other nodes (22,423 and 22,421) at depths of 60–91 m and all other nodes (5–139 m in depth) contributing less than 5% of observations (Fig. 3b and Supplementary Fig. S2, Supplementary Table S1). However, the number of nodes (${n}_{nodes}$) with observations and the relative contribution of nodes in shallow (< 50 m) versus deep (≥ 50 m) water differed through time (Fig. 3c and Supplementary Fig. S3).

**Figure 4:**
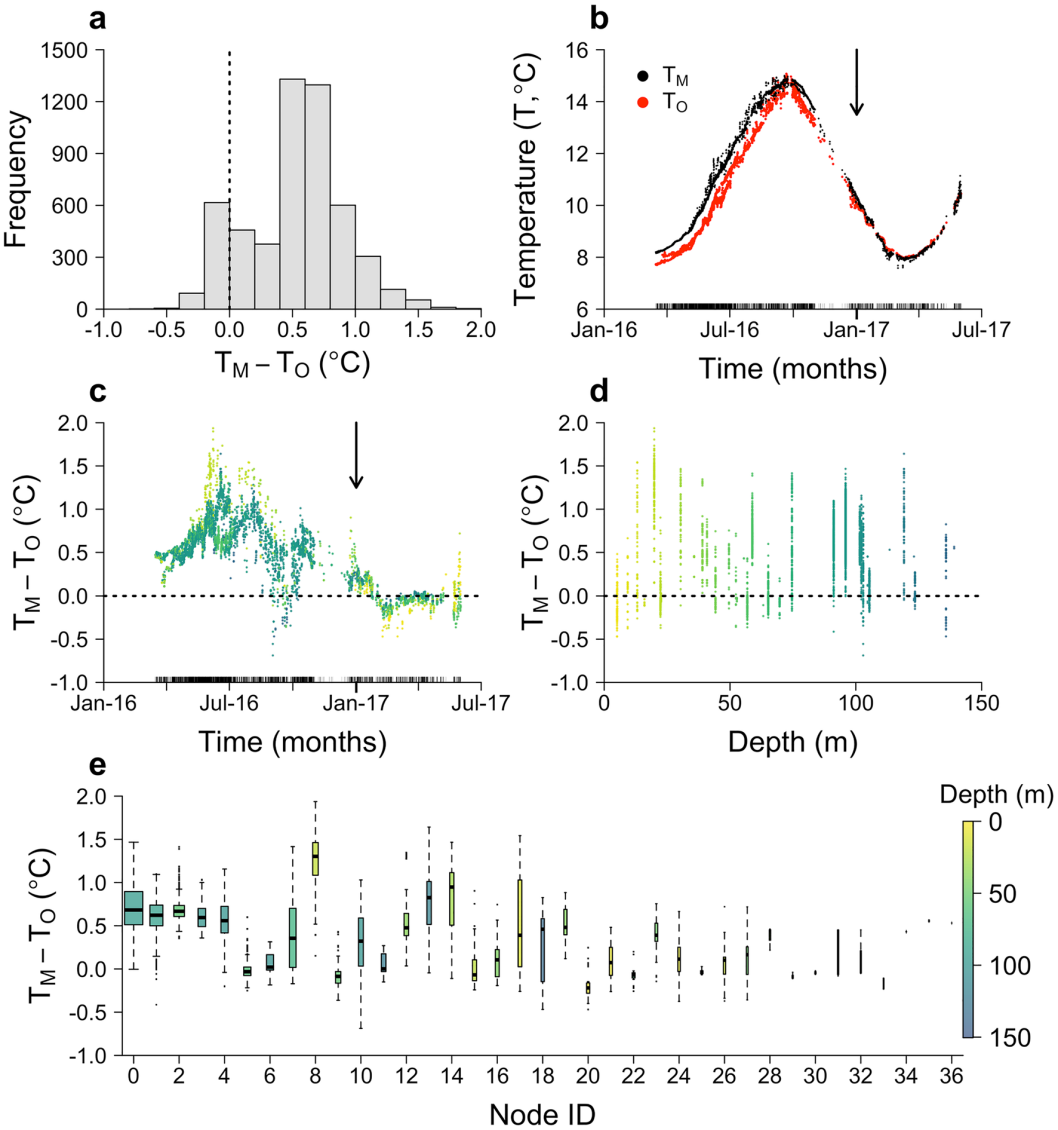
Bottom-temperature validation results. (**a**) The frequency distribution of differences between modelled ($M$) and observed ($O$) bottom temperatures ($T$). The vertical dashed line marks the point of no difference. (**b**) Modelled and observed time series. (**c**) The difference between modelled and observed temperatures through time. In (**b**) and (**c**), the rug marks times with observations and the arrows mark the transition from 2016 to 2017 (when, independently of these observations, the model was upgraded). (**d**) The difference between modelled and observed temperatures in relation to node depth (below mean sea level). (**e**) The difference between modelled and observed temperatures by node (nominally labelled $0,\dots ,36$; see Supplementary Table S1). For each boxplot, the thick black line marks the median, the edges of the box mark the first (${Q}_{1}$) and third (${Q}_{3}$) quartiles and bar ends mark the minimum and maximum values (excluding statistical outliers). Points mark statistical outliers, which are defined as values that are less than ${Q}_{1}- 1.5\times IQR$ or ${Q}_{3}+1.5\times IQR$ respectively, where $IQR$ is the interquartile range. Box width is proportional to the number of observations (see Supplementary Table S1).

Across the whole time series, overall model skill was relatively high (Fig. 4, Table 1). Modelled and observed bottom temperatures were strongly correlated according to Pearson’s Product Moment Correlation Coefficient ($R$ = 0.99) and closely matched according to the Index of Agreement ($d$ = 0.98). The mean difference between modelled and observed bottom temperatures (Mean Bias [$MB$]) was 0.53 °C and the mean absolute difference (Mean Error [$ME$]) was similar (0.55 °C). However, differences ranged between -0.69–1.94 °C (Fig. 4), as reflected by the elevated Root Mean Square Error ($RMSE$) score (0.65 °C).

**Table 1:** Model skill metrics for bottom temperature. Column definitions are as follows: total number of observations ($n$), mean modelled temperature ($\widehat{M})$, mean observed temperature ($\widehat{O}$), modelled standard deviation (${\sigma }_{M}$), observed standard deviation (${\sigma }_{O}$), Mean Bias ($MB$), Mean Error ($ME$), Root Mean Square Error ($RMSE$), Correlation Coefficient ($R$) and Index of Agreement ($d$). Units are °C except for $n$, $R$ and $d$ which are unitless.

$n$	$\widehat{M}$	$\widehat{O}$	${\sigma }_{M}$	${\sigma }_{O}$	$MB$	$ME$	$RMSE$	$R$	$d$
5260	10.84	10.31	2.22	2.12	0.53	0.55	0.65	0.99	0.98

Model skill varied through time (Fig. 4b–c, Supplementary Fig. S4–S7 and Supplementary Tables S2–S4) and space (Fig. 4d–e, Supplementary Fig. S8 and S9 and Supplementary Tables S5 and S6). Examination of the raw differences between modelled and observed temperatures suggested a seasonal trend in model error in 2016 (Fig. 4c), with the median difference increasing from 0.59 °C ($n$ = 1692) in spring (March–May) 2016 to 0.87 °C ($n$ = 1,270) in summer (June–August), before declining to 0.52 °C ($n$ = 1,247) over autumn and winter (September–December). In 2017, model error was lower throughout the period of observations (Fig. 4c), with the median difference varying from 0.05 °C ($n$ = 422) in January–February to − 0.03 °C ($n$ = 623) in March–May and − 0.11 °C ($n$ = 6) in June. Across all months, there were clear correlations between model error, the number of observations and average temperature (Supplementary Table S2). However, the same broad patterns were borne out in a simulation-based analysis of model skill metrics that accounted for trends in the number of observations and average temperature (Supplementary Fig. S4, Supplementary Table S3). For example, monthly ensemble-average $ME$ scores increased from 0.40 °C in March to 1.01 °C in June before declining to 0.31 °C in December 2016 and 0.01–0.10 °C in January–June 2017. Over 2016, these errors exceeded the median daily range in modelled bottom temperatures but generally remained below the maximum daily range (Supplementary Fig. S5). Decomposing seasonal trends in ensemble-average skill scores by depth showed that seasonal trends in shallow (< 50 m) water were stronger than deep (≥ 50 m) water, notwithstanding limited data (Supplementary Fig. S6 versus S7). Nevertheless, the influence of shallow nodes on the overall seasonal trend was small: for example, from March–June 2016, ensemble-average $MB$ scores differed by < 0.1 °C depending on whether or not shallow nodes (${n}_{nodes}$ = 3–4) were included in the analysis. Between 2016 and 2017, the comparison of ensemble-average skill scores for March–May inclusive (the period with overlapping observations), revealed strong improvements in $MB$, $ME$, $RMSE$ and $d$ following the update to the model’s boundary forcing (Table 2, Supplementary Table S4).

**Table 2:** Average percentage improvements in ensemble-average skill scores ($P$). Percentages are shown for the Mean Bias ($MB$), Mean Error ($ME$), Root Mean Square Error ($RMSE$), Correlation Coefficient ($R$) and Index of Agreement ($d$). For full details, see Supplementary Table S4.

Metric	$P$ (%)
$MB$	108.53
$ME$	86.13
$RMSE$	84.77
$R$	− 28.85
$d$	94.79

Over space, the visual analysis of differences between modelled and observed temperatures indicated variation in model skill among nodes (Fig. 4d–e). Correlation coefficients indicated that this variation was partly attributable to the number of observations and average temperature (Supplementary Table S5), but there was no clear influence of depth (Fig. 4d). The simulation-based analysis of model skill metrics demonstrated relatively high model skill at most nodes, with ensemble-average skill scores for $MB$, $ME$ and $RMSE$ broadly below 0.5 °C and $R$ close to one, although ensemble-average $d$ scores were more variable (Supplementary Fig. S8, Supplementary Table S6). There was no clear clustering of ensemble-average skill scores in space or by depth (Supplementary Fig. S9). Across the study site at large, spatial variation in modelled bottom temperatures was limited but sufficiently high relative to model skill metrics to indicate that our results cannot be widely generalised across the study site at this stage (median IQR in modelled bottom temperatures across the site = 0.39 °C, Supplementary Fig. S10).

### Temperature-depth profile validation

For the validation of modelled temperature-depth profiles, 26 recreational angling events were identified, comprising five events during individuals’ time at liberty and 21 tag retrieval events (Supplementary Table S7). A sample of eight temperature-depth profiles, comprising 64 observations in total, were associated with valid coordinates and used for validation. These profiles were derived from angling events that lasted 8–20 min. During this time, the impact of tidal elevation on model layer depth is expected to have been minimal, given a median absolute change of 0.19 m per hour, demonstrating that the interpolation method used to assemble comparable modelled profiles was appropriate. In terms of temporal validation effort, observations were collected on four different hours on eight different days from August 2016–April 2017. Spatially, observations were collected from eight unique locations corresponding to five nodes (Fig. 1 and Supplementary Fig. S1). Observations spanned a depth range of 0–163 m and a temperature range of 7.81–14.05 °C.

**Figure 5:**
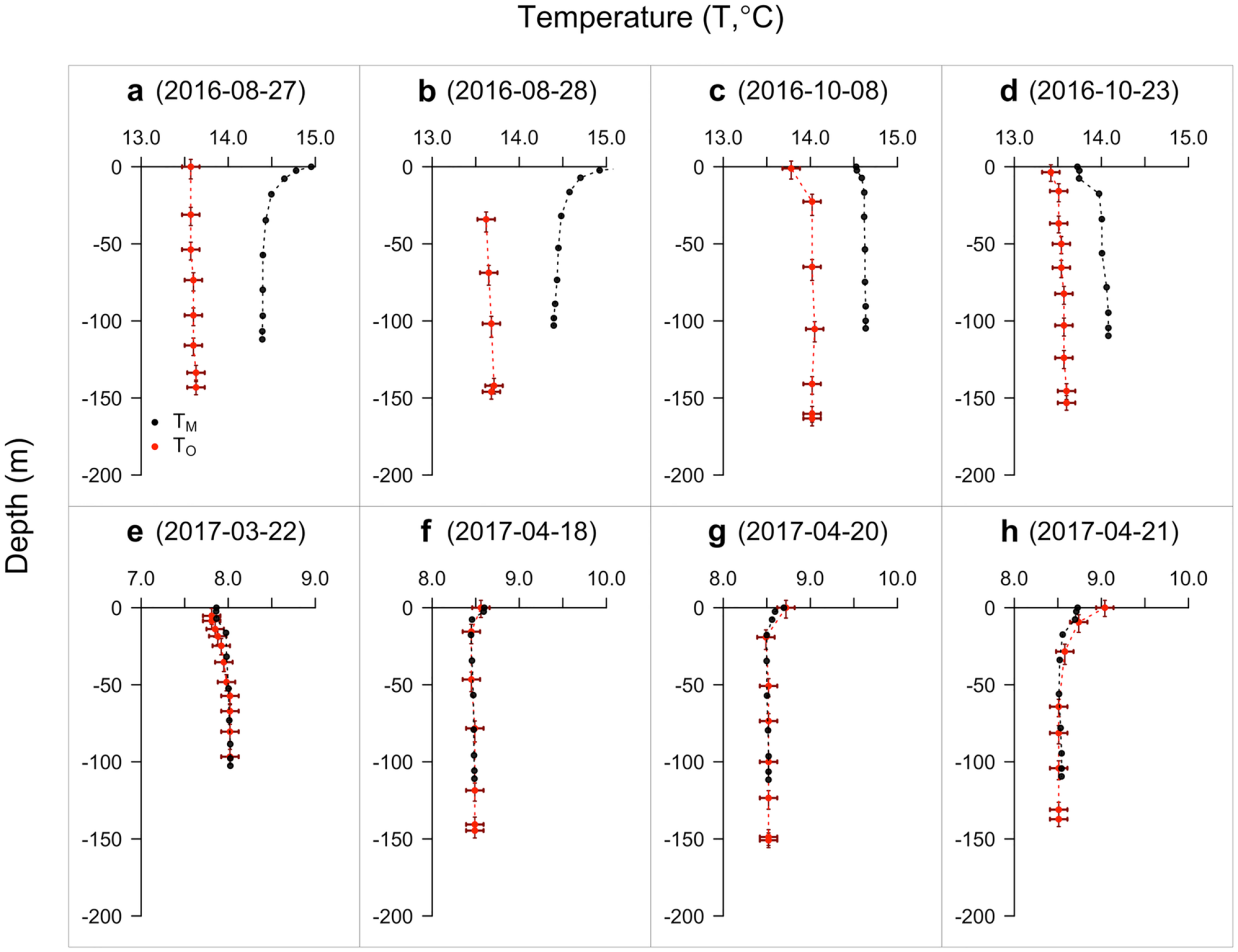
Temperature-depth profile validation. Each panel (**a–h**) shows a modelled (black) and observed (red) temperature-depth profile for a particular date. For profile **b**, near-surface temperature observations were not recorded. Note that the temperature axis differs among panels, but the range is constant (2 °C), so the panels are comparable. Differences between the depth of the seabed layer in the model and the maximum depth of individuals reflect local variation in bathymetry that is not resolved by the model mesh. Error bars mark uncertainty in temperature observations and the depths to which these correspond.

The results from the visual comparison of modelled and observed temperature-depth profiles were largely consistent with the results for bottom temperature (Fig. 5). In all cases, modelled temperatures were generally within 1.5 °C of observations. For the four profiles from 2016 (obtained in August and October), modelled temperatures exceeded observed temperatures across all depths (Fig. 5a–d). This overprediction was stronger for the two profiles in August and particularly noticeable near the surface where, in contrast to relatively linear observed profiles, modelled temperatures increased by 0.5 °C in the upper 10–20 m (Fig. 5a–b). In October, the shape of the observed profiles was captured more effectively by the model, with both observed and modelled temperatures marginally cooler near the surface at this time (Fig. 5c–d). In 2017, all four samples (collected in March and April) were accurately modelled (Fig. 5e–h), although there was a small discrepancy in the near-surface predictions for the final temperature-depth profile (Fig. 5h).

## Discussion

This study provides a unique demonstration of the use of benthic animal-borne sensors and citizen-science data for the opportunistic, empirical validation of an ocean model. In Scotland, the dataset assembled in this study for bottom-temperature validation contributes towards a key data gap. In line with previous research^29,30^, the data provide robust confirmation that modelled bottom temperatures are relatively accurate (typically within ± 1 °C) but were previously consistently biased (mean overprediction = 0.53 °C). However, an independent update to the ocean model’s external forcing system substantially improved modelled bottom temperatures. Temperature-depth profile data are more limited, but available observations suggest that the results for bottom temperatures may apply throughout the water column. This work demonstrates the potential contributions of benthic animal-borne sensors for oceanographic data collection in benthic environments that are otherwise difficult and expensive to sample, extending previous studies that have principally focused on satellite-telemetered seabirds^13,18,19,21,22^ and diving seals^12,24,25^. Given the range of species studied using archival tags and passive acoustic telemetry in aquatic enviroments^2–4^, there are substantial opportunities to develop this line of work to support research in movement ecology and oceanography in a wide range of settings.

The key result of this study for WeStCOMS is that modelled (bottom) temperatures are resolved relatively accurately across model nodes and an independent model update, designed to improve the match between glider data from the model’s boundary and modelled temperatures, also improved skill deep within the model’s interior. As expected, in 2016 model skill was lower in summer (especially in shallow water) when warmer temperatures were associated with increased diurnal and weekly variability; yet this trend remained apparent after accounting for the change in average temperature. Given available data, the explanation(s) for this trend remain(s) uncertain. One possibility is that flapper skate exploit fine-scale variation (not captured by the model mesh) in temperature for activities such as resting, which is thought to increase during summer when vertical activity is lower^43^ and known to occur in cooler-than-average habitats in other elasmobranchs^44^. Another possibility is a seasonal bias in the influence of the model’s temperature forcing in the study site, but further data are required to evaluate this possibility. In line with limited data, we cannot generalise our findings to other areas, times or model parameters, but the analysis of spatial variation around areas with receivers suggests that our results may be broadly applicable across the central portion of the study site, although variation in modelled temperatures across the study site more widely limits further generalisation at this stage.

The results for bottom-temperature validation in this study depend on the assumption that flapper skate are benthic animals. This assumption is consistent with skate morphology and diet studies of the common skate species complex (*D. batis*), which includes flapper skate and the common blue skate (*D. batis*)^45^, that have revealed the exploitation of a wide range of benthic prey^46–48^. The way that flapper skate ‘dig’ into the sediment when hooked by recreational anglers also demonstrates benthic movements^40^. Moreover, recent research has shown that it is possible to reconstruct movement trajectories for flapper skate under the assumption that they remain near to the seabed (Lavender et al., in prep). In some situations, burial within the sediment, as documented in other species^49^, or propulsion above the seabed, as hypothesised to account for the presence of pelagic prey in the stomachs of common skate^47^, may induce limited discrepancies between observed and modelled bottom temperatures, but quantifying their influence requires further research.

The other main assumption of the validation method developed in this study is that nearest neighbour interpolation in time and space is appropriate. In many cases, this choice is likely to be a pragmatic option, given the accuracy of observations, the magnitude of spatiotemporal variation and research objectives. In our study site, the detection range around receivers (used to localise individuals) has been estimated as approximately 425 m, in line with many other passive acoustic telemetry systems^50^. This translates into a location accuracy of approximately 0.57 km^2^ for detected individuals, which broadly aligns with the resolution of the model mesh in the study site. Together with the accuracy of temperature records on archival tags (0.1 °C) and the variation in modelled bottom temperatures between neighbouring nodes (median absolute difference = 0.005 °C), this suggests that nearest neighbour interpolation in space for bottom-temperature validation was sufficient. Similarly, given the accuracy of archival temperature records compared to the magnitude of temporal variation in temperatures (median absolute hourly difference = 0.010 °C), it is clear that nearest neighbour interpolation in time was also sufficient. For the temperature-depth data derived from recreational angling, location records are similarly uncertain, given that anchored charter vessels can drift across an area spanning several hundred square metres as a result of the effects of the tide. During ascent, continued change in tidal elevation (median change = 0.185 m per hour) implies a small discrepancy between the actual depth of the layers and the depth of the layers calculated given the tidal elevation at the nearest hour to the onset of the angling event, but this is negligible in the context of uncertainty in the depths of tagged individuals (± 4.77 m) and small-scale variation in the depth of the seabed (unresolved by the model mesh). Nevertheless, in other settings in which animal location and temperature/depth data are more accurate, our methods would be enhanced by the use of a refined three-dimensional interpolation method based on the Finite Volume flux solution.

Despite the limitations of this study, taken together with previous research^29,30^, the collective evidence that temperatures are resolved accurately (typically within ± 1 °C) in WeStCOMS is strong. For flapper skate, this result confirms the validity of previous analyses of detection patterns in relation to temperature^39^, and supports the use of modelled temperatures in other settings^29,30^. More broadly, data collected from flapper skate could support the validation of other unstructured-grid FVCOM implementations, such as the Scottish Shelf Model^51^, alongside structured-grid regional hydrodynamic models, such as the North-West European Shelf Atlantic Margin Model (AMM15)^52^. Moving forwards, improvements in the availability of in situ data, the resolution and quality of remote-sensing data products and the parent ocean model used for the boundary forcing should support further improvements in WeStCOMS skill and other nested regional ocean models. However, model validation remains an active area of research, especially for conditions near the seabed such as bottom-current velocities where validation datasets remain limited^29,30^.

We anticipate that there are widespread opportunities to extend our methods and expand the range of animal oceanographers in aquatic environments. Passive acoustic telemetry is widely deployed in aquatic systems, including as part of research on other skate species^27^ and pelagic species^3,8^. In many systems, multi-sensor acoustic tags are used that transmit acoustic (location) and sensor (oceanographic) information to receivers^2^, streamlining the integration of datasets required for model validation. Fine-scale acoustic positioning systems that implement multilateration (hyperbolic positioning) improve location accuracy are also growing in popularity^53^. Alongside acoustic telemetry, archival tags are widely deployed^2,27^. In our study site, the recapture rate (21/40 archival tags in this study) is unusually high, but even in other systems standard archival tags and pop-up satellite archival tags provide a means to collect extensive temperature-depth time series^2,27^. At the same time, multi-sensor capacities provide a means to derive information on other oceanographic fields^8^. Following detachment, pop-up tags are effectively passive drifters whose landing locations can be predicted by ocean models to support tag recovery and test model skill, as demonstrated for telemetry collars dropped by polar bears^15^. These opportunities are not a panacea in oceanography. Data from tagged animals are subject to welfare concerns, constrained by biases^10^ and do not reduce the need for comprehensive and sustained ocean observing systems. For benthic taxa, data transmission speeds are an additional challenge that significantly constrain the potential contributions of animal-borne sensors to coastal oceanographic networks. For this reason, at least in the near future, it seems likely that the contributions of benthic animal-borne sensors will be restricted to model validation. Yet despite these caveats, where animals have been tagged as part of research on their movements and management, there is a strong case to maximise their uses. Given that the volume of animal movement data is likely to continue to increase in the future, a coordinated framework for collating and exploiting these data in operational oceanography would be valuable^9,10^.

Citizen-science programmes have substantial potential to support this line of research, but to date many projects have focused on the collection of biodiversity datasets rather than information on the physical ocean environment^16,54^. This study provides a clear demonstration of the potential contributions of recreational anglers to interdisciplinary research in movement ecology and ocean modelling. In our study site, the work of Scotland’s nature agency (NatureScot) and partner institutions to strengthen relationships between charter skippers, anglers and scientists has been particularly important. In this study, relationships with skippers facilitated tag deployment and the wider dissemination of research in the recreational angling community. Specific actions to support recreational mark-recapture angling (such as the provision of passive integrated transponder tags and scanners to skippers) and the establishment of a photo-identification database (‘SkateSpotter’) have also helped to connect anglers with scientific research and provided a means for anglers to submit and receive data^37,42^. Angler mark-recapture records recorded on SkateSpotter from individuals’ time at liberty, alongside the return of tags from recaptured skate by recreational anglers, underpinned the work presented here alongside previous studies^36,38–41,43^, and partnerships between NatureScot, researchers and other stakeholders continue to support ongoing research on skate in the region^55,56^. This demonstrates the value of fostering positive relationships between stakeholders and the important contributions that recreational anglers can make to scientific research. Given the prevalence of recreational angling and animal tagging in coastal marine environments, we anticipate that there may be opportunities to expand these contributions in many settings^16,57,58^.

This line of research has important implications for oceanography, ecology and marine management. There have long been calls for ‘coastal observatories’ designed to collect operational oceanographic data systematically for research, policy and regulation^16,17^. In a regulatory context, for example, data are required to support licensing and consenting processes associated with strategic and environmental impact assessments. Yet comprehensive and sustained observations in coastal systems remain sparse, particularly for near-seabed variables such as bottom temperature^16,17^. In this context, data from animal-borne sensors can make an important contribution to operational oceanography, supporting ocean observing systems^9,10,12^, modelling^12,13^ and regional ocean model validation, as shown here and elsewhere^14,15^, with implications for management. For example, in fjordic countries such as Scotland, a key area of interest in the use of regional ocean models lies in the development of early warning systems for harmful algal blooms and sea lice dispersal because of the impacts on fin-fish aquaculture^29,30^. Water temperature fluctuations can shape the emergence of harmful algal blooms^59^ and the accuracy of early warning systems thus hinges upon the accuracy with which models reproduce temperature profiles, alongside other factors. By quantifying the accuracy of model predictions, validation studies help to guide model refinements and develop confidence in the use of model outputs for sustainable marine management^29,30^.

In conclusion, this study demonstrates the successful use of benthic animal-borne sensor data for ocean model validation. In Scotland, the data collected from flapper skate in association with recreational anglers have helped to address a key data gap and provided a unique opportunity to validate WeStCOMS, but could also support the validation of other models^51,52^. This work highlights opportunities to strengthen links between research disciplines and stakeholders in other systems to expand the range of animal oceanographers in marine environments and support interdisciplinary research in ecology, oceanography and management.

## Methods

### Study site

The west coast of Scotland is a complex environment characterised by sea lochs, peninsulas and narrow channels (Fig. 1). WeStCOMS represents this area with a mesh comprising 46,878 nodes (see Supplementary Information Sect. 1). There are 11 terrain-following sigma-coordinate layers (layer 1 is at the surface and layer 10 is at the seabed) that rise and fall with the tides. Within the study site, the Loch Sunart to the Sound of Jura MPA occupies 741 km^2^, within which the 5,055 cells (around nodes) each span a median area of 0.13 km^2^. The bathymetric environment includes shallow-water (< 50 m) platforms alongside channels and basins up to 290 m in depth^60^. Bottom and sea-surface temperatures vary from a minimum of approximately 6 °C in winter to a maximum of 16 °C in late summer. Over the summer, thermal stratification (1–2 °C in magnitude) develops in the upper (< 100 m) water layers.

### Electronic tagging and tracking

A passive acoustic telemetry array comprising 58 Vemco 69 kHz receivers was deployed from March 2016–July 2017 in the MPA to study skate movement (see Lavender et al.^39^ for full details). Previous studies have estimated the detection range (here defined as the distance from a receiver at which detection probability is 0.5) as 425 m^39,61^. This translates into a surveyed area of approximately 0.56 km^2^ per receiver.

Within the MPA, forty skate were captured and tagged with acoustic and archival tags^39^. Skate were captured from charter vessels using baited lines with barbless hooks. Each skate was tagged with a Vemco V13 or Thelma Biotel MP-13 miniature (13 × 25 mm) coded acoustic transmitter, programmed with a nominal transmission delay of 60 s, on the leading edge of the right wing. The transmission delay was randomised to minimise the probability of transmission collisions which can cause detection failure or false detections^62^. Each skate was also tagged with a Star Oddi Milli-TD archival tag on the leading edge of the left wing. Archival tags were programmed to record pressure (depth) to a resolution of 0.24 m and an accuracy of 4.77 m, and temperature, to a resolution of 0.032 °C and an accuracy of 0.1 °C (according to manufacturer specifications), every two minutes during deployment^43^ (see Supplementary Information Sect. 2).

Capture and tagging were approved by the ethics committee of the University of St Andrews (number SEC21024). All regulated procedures involving animals were carried out in compliance with The Animals (Scientific Procedures) Act 1986 under the Home Office Project License number 60/4411 by competent Personal License holders.

Following tagging, individuals were released. During their time at liberty, acoustic transmission codes were detected by receivers when individuals moved within range. (Other information, such as ‘time to arrival’, depth or temperature was not recorded by receivers.) Meanwhile, depth and temperature observations were recorded by archival tags. Data from 21 archival tags have been recovered from skate recaptured by recreational anglers to date. These data were processed and made available by previous studies^39–41,43^ for this work.

### Validation datasets

Within the movement datasets collected from skate, two sources of validation data for WeStCOMS were identified. The first source of data comprises bottom-temperature observations collected by archival tags during time spent on the seabed (i.e., undisturbed activity). The second source of data comprises temperature-depth profiles sampled by archival tags during recreational angling events (including events that occurred during individuals’ time at liberty and tag retrieval events). These data were used to validate modelled bottom and water-profile temperatures at times when the location of observations could be identified from passive acoustic telemetry or mark-recapture records (see Supplementary Information Sect. 2).

### Bottom-temperature validation

Data processing and analyses were implemented in R (version 4.0.2)^63^. To validate modelled bottom temperatures, a dataset with observed and modelled temperatures was assembled using the fvcom.tbx package^64^. Observed data comprised temperature records on archival tags when individuals were simultaneously detected at receivers. Only detections that passed the short interval criterion for false detections^62,65^ were considered in this analysis^39^. For each detection, we simply assumed that the detected individual was on the seabed near to the receiver (typically within 425 m), given the absence of more precise information on location, and identified the nearest node. (Archival depth records were not used to localise skate on the seabed more accurately in this analysis because multiple locations around each receiver typically matched (or were close to) the individual’s observed depth.) For each node, on each hour of the detection time series we calculated the mean observed temperature and derived corresponding temperature predictions (for the same node, hour and the tenth depth layer) using nearest neighbour interpolation (see Supplementary Information Sect. 3.1).

Using the validation dataset, we quantified validation ‘effort’ and model skill. Effort was quantified in terms of the number of observations though time and space. Overall model skill was quantified using five standard regression metrics:

- **A** Correlation Coefficient ($$R$$).
- **B** Index of Agreement ($d$).
- **C** Mean Bias ($$MB$$).
- **D** Mean Error ($$ME$$).
- **E** Root Mean Square Error ($RMSE$).

(See Supplementary Information Sect. 3.2 for metric definitions.)

We also examined variation in model skill over time and space by (a) visualising the differences between modelled and observed temperatures (i) through time, (ii) by node and (iii) in relation to depth and (b) evaluating model skill metrics for (i) each month and (ii) each node. In these analyses, we were specifically interested in evidence for systematic variation in model skill through time or space (i.e., by node). We expected that temperature variability would increase (and model skill would decrease) with sample size and average temperature and we confirmed this expectation in a correlation analysis of summary statistics for these variables calculated for each month and node. Therefore, for the analysis of model skill metrics (b), we used a five-stage stratified random sampling algorithm to account for variation in sample size and average temperature:

- **A** *Selection* We considered nodes/months with at least $n$ = 5 observations (the 25^th^ percentile).
- **B** *Sampling* For each node/month, we randomly sampled $n$ = 5 observations/predictions, from which model skill metrics were calculated. Alongside the metrics mentioned above, in this analysis we also considered the normalised $MB$, the normalised $ME$ and the normalised $RMSE$ to account for variation in average temperature (see Supplementary Information Sect. 3.2).
- **C** *Iteration* We repeated the sampling step for $n = \mathrm{1,000}$ simulations to generate a distribution of model skill scores for each node/month and metric.
- **D** *Averaging within categories* For each metric, average skill scores were calculated for each node/month as the median score across all simulations.
- **E** *Averaging across categories* For each metric, ensemble-average skill scores were calculated for (i) each month (as the mean average skill score across nodes for each month) and (ii) node (as the mean average skill score across months for each node). Both sets of ensemble-average skill scores were visually inspected to examine variation in model skill. Since ensemble-average skill scores are based on average skill scores from simulations with a fixed number of samples, they are robust to changes in the number of observations for each node/month, but they may be influenced by changes in the specific nodes or months with observations (for example, the proportion of nodes with observations in shallow [< 50 m] versus deep [≥ 50 m] areas). Therefore, in the analysis of monthly ensemble-average skill scores (i), we separated trends for shallow versus deep nodes. We anticipated that nodes in shallow water would experience a stronger seasonal trend in model skill and we quantified their influence on overall patterns by comparing ensemble-average skill scores calculated across all nodes versus only those nodes in deep water. Monthly ensemble-average skill scores across all nodes were compared for the period with overlapping data (March–May) in 2016 and 2017 to evaluate the percentage improvement in skill attributable to the update to the model forcing in January 2017. In the node-based analysis (ii), we analysed variation among nodes similarly and quantified the variation in ensemble-average skill scores in relation to depth using linear regression. For both sets of ensemble-average skill scores, we contextualised estimates of model skill with estimates of the magnitude of spatiotemporal variation in modelled bottom temperatures across the study site (see Supplementary Information Sect. 3.3).

### Temperature-depth profile validation

Temperature-depth profiles recorded during recreational angling events by tagged individuals that were pulled to the surface were used to validate modelled temperature-depth profiles. Angling events were identified in the mark-recapture database maintained by NatureScot, Marine Scotland Science and the Scottish Association for Marine Science^40^ (see Supplementary Information Sect. 4.1). Each angling event was defined from the time of the last depth observation preceding the capture ascent to the time of the first observation at the surface^40^ (Fig. 2). For each profile, observational uncertainty in depth and temperature was quantified from manufacturer specifications (see Supplementary Information Sect. 4.2). Using these data, validation ‘effort’ was quantified in terms of the number of observations and their spatiotemporal distribution.

For each angling event, modelled temperatures for each depth layer in the model were obtained for the nearest node and hour (from the start of the angling event). Model layer depth was calculated, accounting for modelled tidal elevation at the start of the angling event, using the fvcom.tbx package^64^ (see Supplementary Information Sect. 4.3).

Modelled temperature-depth profiles and observed profiles from archival tags were visualised together for each angling event to examine model skill. However, given the mismatch between the depths at which temperatures were observed and the depths of the sigma-coordinate layers in the model, alongside limited data for this analysis, model skill metrics were not calculated.

## Supplementary Information

Supplementary Information 1.

Supplementary Information 2.

Supplementary Information 3.

## Data Availability

R code and the validation datasets are available in the westcoms_validation GitHub repository (https://github.com/edwardlavender/westcoms_validation).
