# Supplementary material for: Benthic animal-borne sensors and citizen science combine to validate ocean modelling
Source: Sci Rep. 2022 Oct 5;12:16613. doi: 10.1038/s41598-022-20254-z (PMC9534998; doi:10.1038/s41598-022-20254-z)
Supplement: Supplementary file 1 — Supplementary Information 1. [file 41598_2022_20254_MOESM1_ESM.pdf]

Supplementary figures

Benthic animal-borne sensors and citizen science combine to validate ocean modelling

Edward Lavender[1,2*], Dmitry Aleynik[3], Jane Dodd[4], Janine Illian[5], Mark James[2], Sophie Smout[1,2,7], James Thorburn[2,6,7]

[1]Centre for Research into Ecological and Environmental Modelling, University of St Andrews, St Andrews, United Kingdom

[2]Scottish Oceans Institute, University of St Andrews, St Andrews, United Kingdom

[3]Scottish Association for Marine Science, Oban, United Kingdom

[4]NatureScot, Oban, United Kingdom

[5]School of Mathematics and Statistics, University of Glasgow, Glasgow, United Kingdom

[6]School of Biological Sciences, Queen's University Belfast, Belfast, United Kingdom

*** Correspondence:**

Edward Lavender

el72@st-andrews.ac.uk

[7]These authors jointly supervised this work.

Supplementary figures

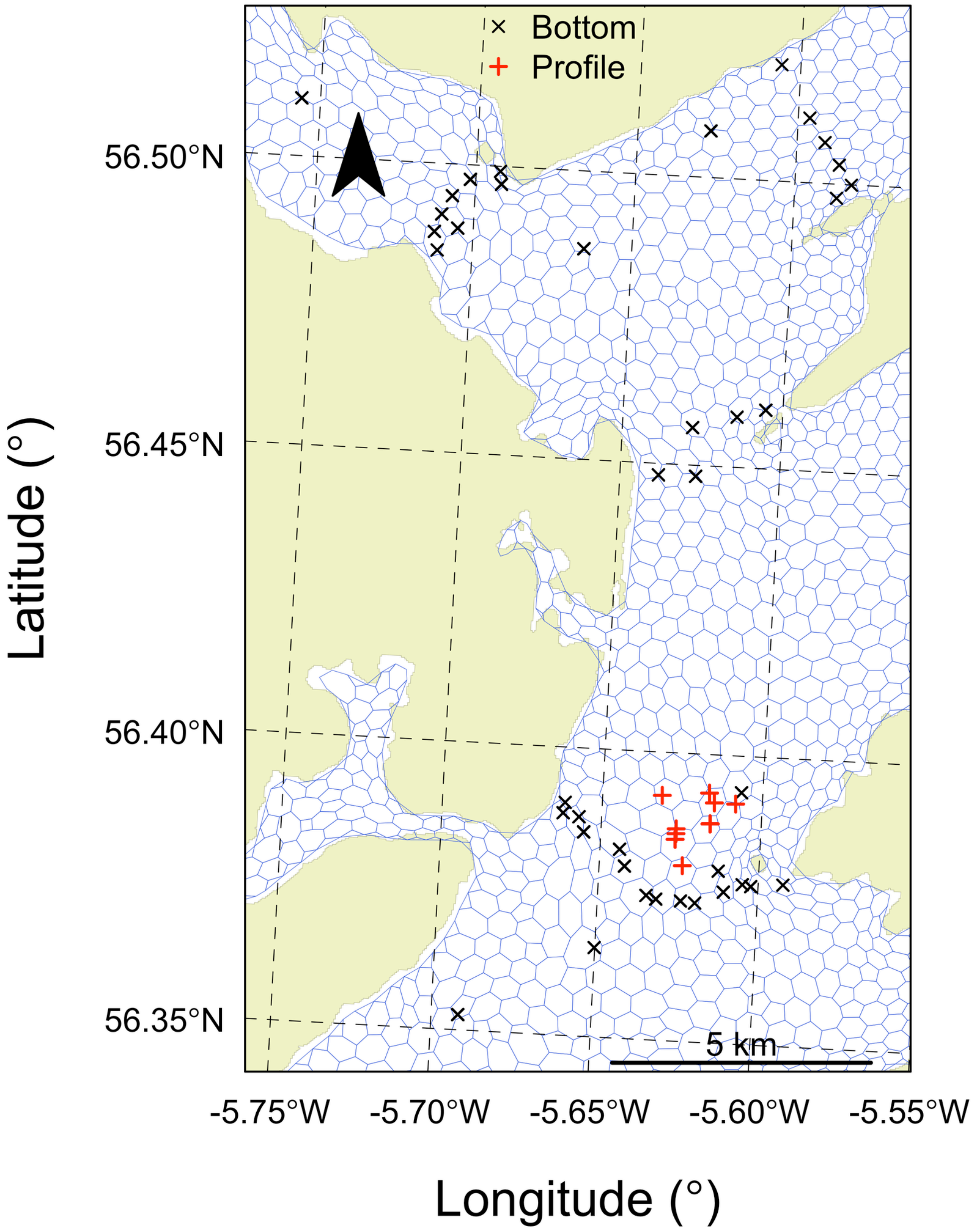

Figure S1. A zoom-in of the study site. Following Figure 1, the mesh around nodes (connecting the elements in the native triangular mesh) is shown in blue. Acoustic receivers that recorded detections associated with bottom-temperature observations recorded by archival tags are marked in black (n = 40). Angling locations associated with temperature-depth profiles are marked in red (n = 8). The coordinate reference system is British National Grid and the north arrow points towards grid north. Gridlines mark lines of longitude and latitude. Bathymetry data were sourced from Digimap © Crown copyright and database rights [2019] Ordnance Survey (100025252) and coastline data were sourced from the Database of Global Administrative Areas.

Supplementary figures

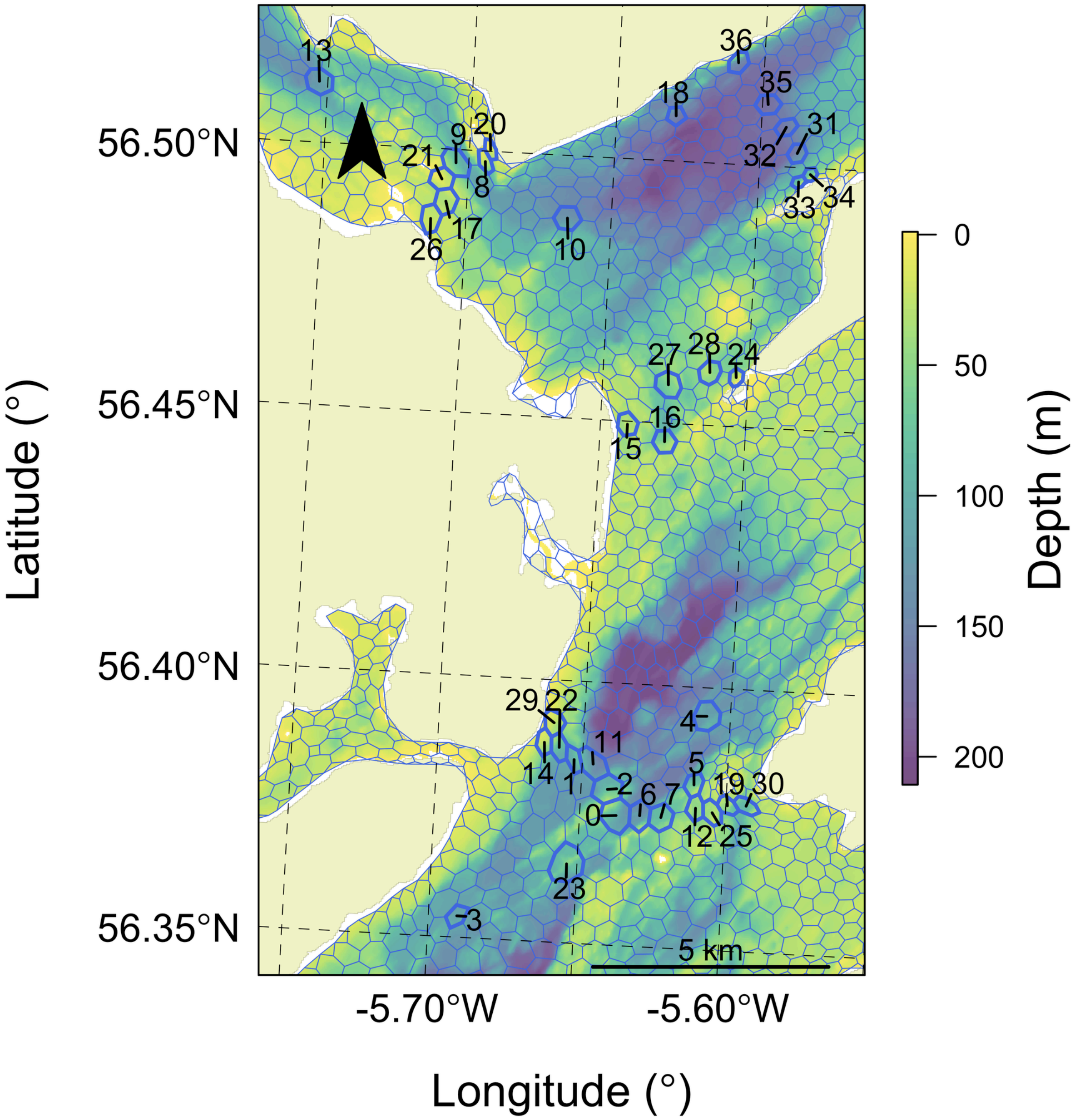

Figure S2. Bottom-temperature validation effort across space. Nodes with observations (n = 37) are ranked from 0–36 in order of the number of observations from most to fewest (see Table S1). The model mesh (around nodes) is shown in blue. The bathymetric depth is shown at 1 arc-second resolution. Note that the bathymetric depth varies at a scale smaller than that captured by the model mesh. Map properties follow Figure S1.

Supplementary figures

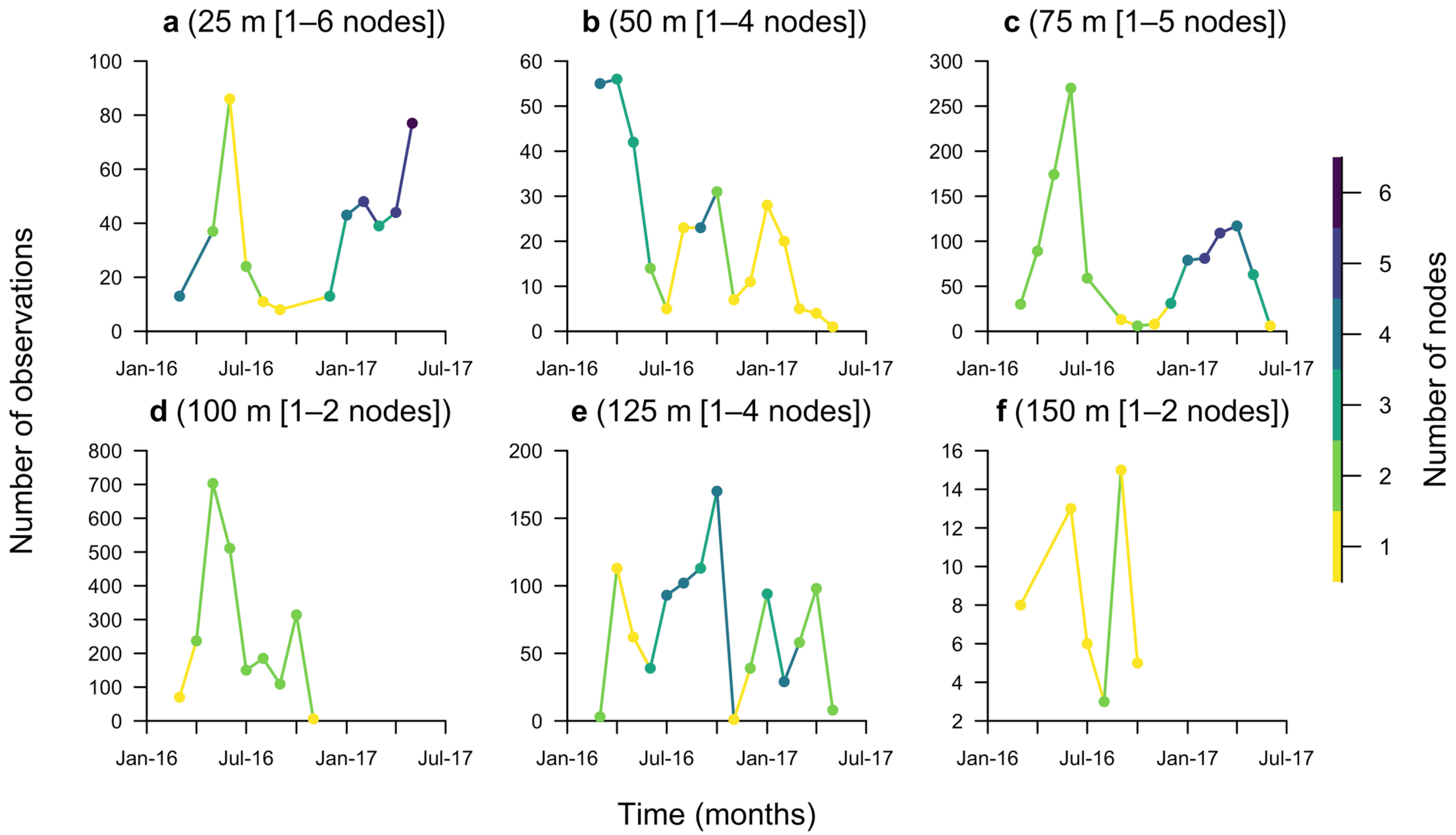

Figure S3. Bottom-temperature validation effort by depth through time. Each panel (**a–f**) shows the total number of validation observations across all nodes with observations in each 25 m depth bin per month. Panel titles denote the maximum depth of each depth bin along with the range in the number of nodes with observations (over the period with observations).

Supplementary figures

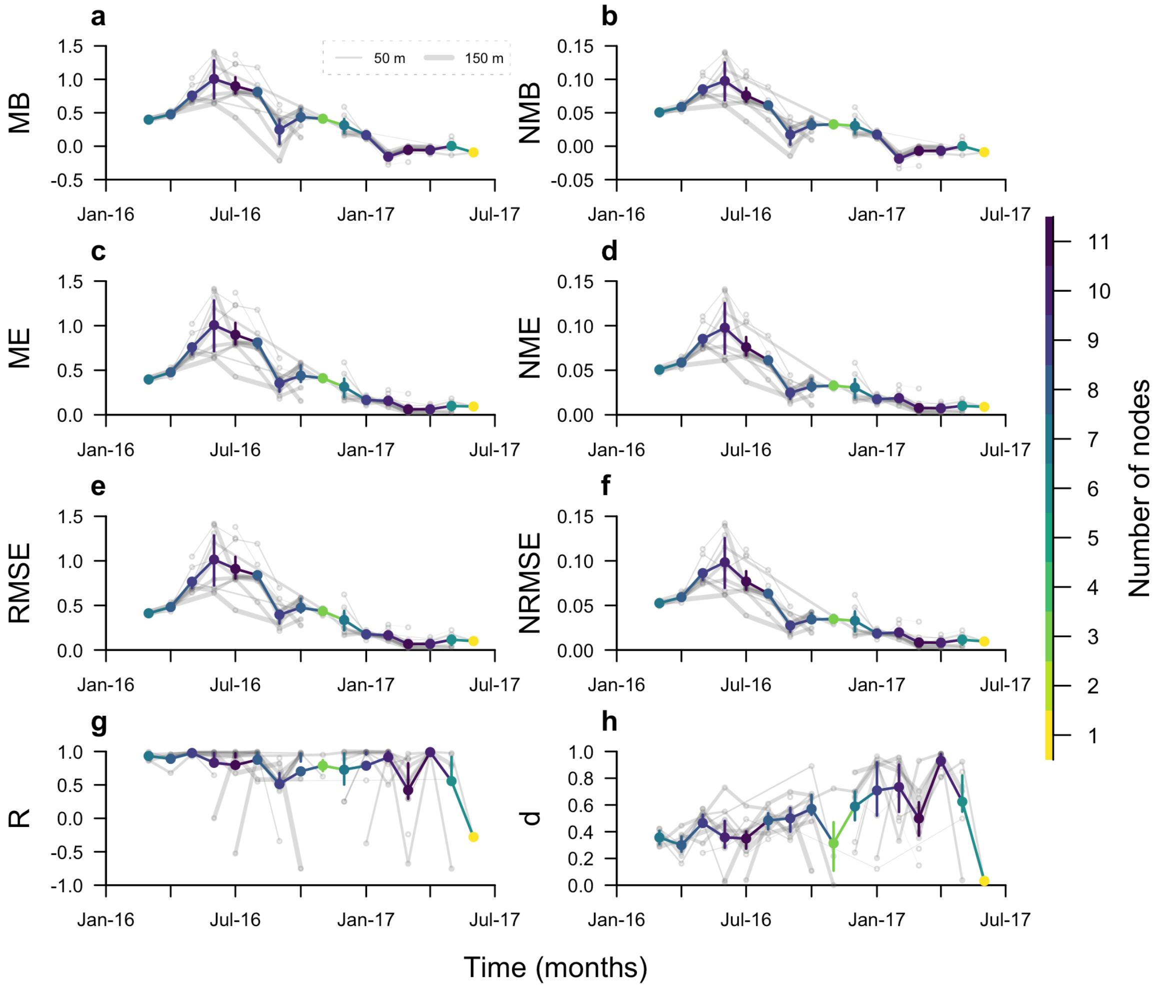

Figure S4. Bottom-temperature model skill metrics by month. Panel metrics are as follows: **a,** Mean Bias; **b**, Normalised Mean Bias; **c,** Mean Error; **d,** Normalised Mean Error; **e**, Root Mean Square Error; **f,** Normalised Root Mean Square Error; **g,** Correlation Coefficient; and **h,** Index of Agreement. In each panel, the coloured points and line indicate the ensemble-average skill score in each month. Vertical error bars show the interquartile range in average skill scores across analysed nodes in each month; note that these do not always align with the mean. Average skill scores are shown in grey. The points mark monthly values and line thickness is a continuous measure of node depth (as shown in **a**). Note that not all analysed nodes had sufficient observations to calculate average skill scores in each month. Units for **a**, **c** and **e** are °C; other metrics are unitless. Point colour corresponds to the number of nodes with observations.

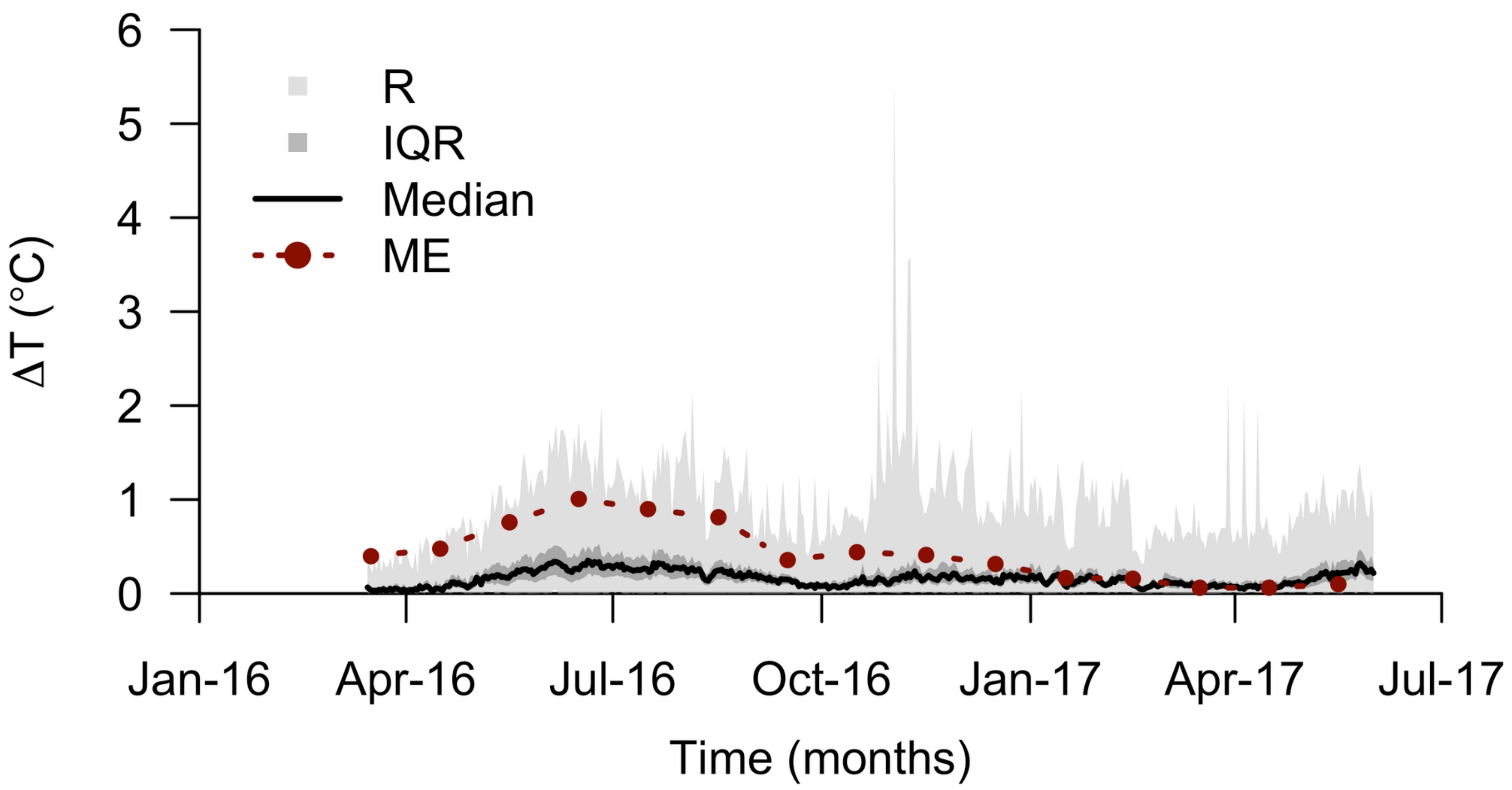

Figure S5. Bottom-temperature ensemble-average Mean Error (*ME*) scores by month in relation to daily bottom-temperature ranges. The red line marks the *ME* (centred in the middle of each month); the black line marks the median daily temperature range; and the dark and light grey envelopes mark the interquartile range and the range in daily temperature ranges across all nodes in the Loch Sunart to the Sound of Jura Marine Protected Area.

Supplementary figures

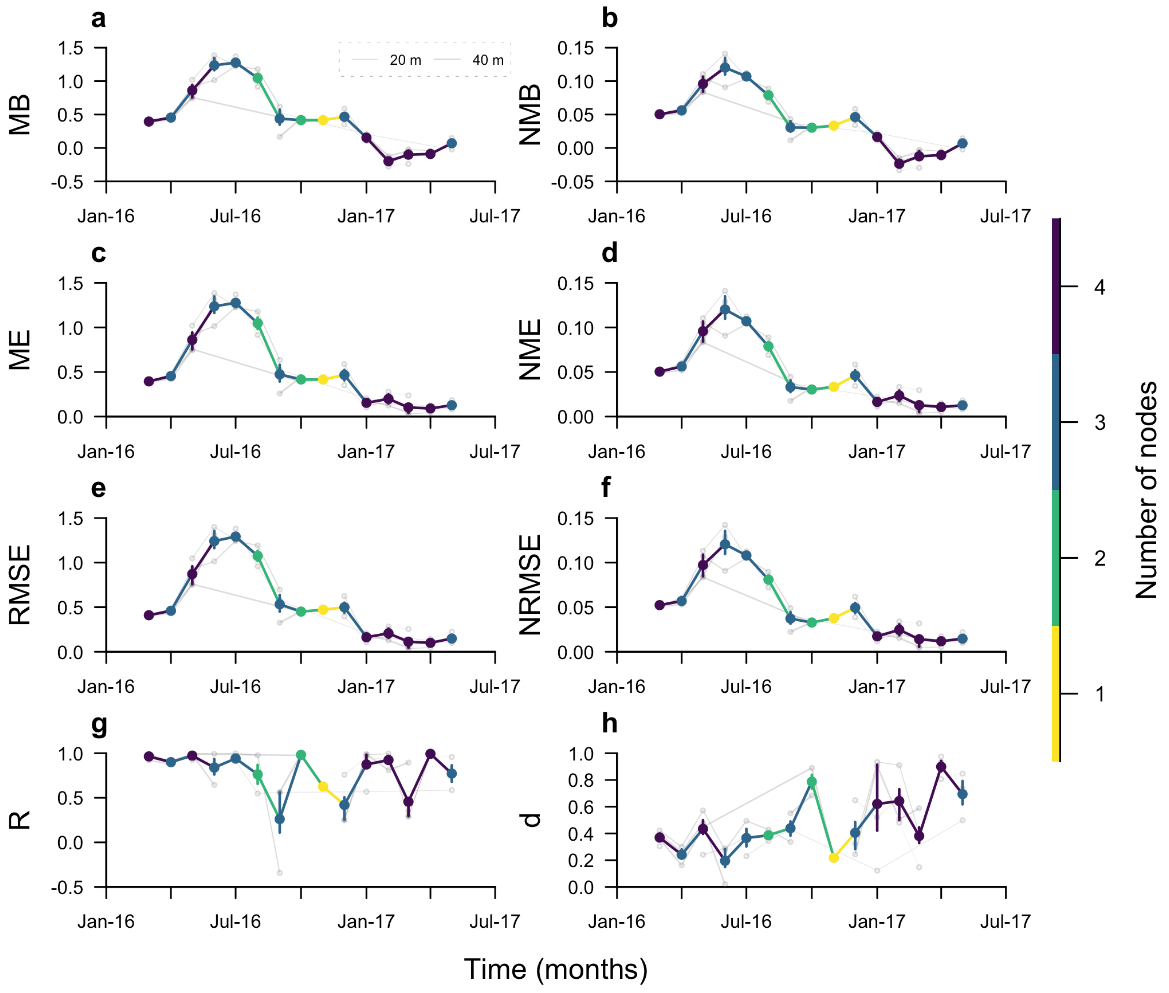

Figure S6. Bottom-temperature model skill metrics by month for nodes in shallow (< 50 m) areas. Panels follow Figure S4.

Supplementary figures

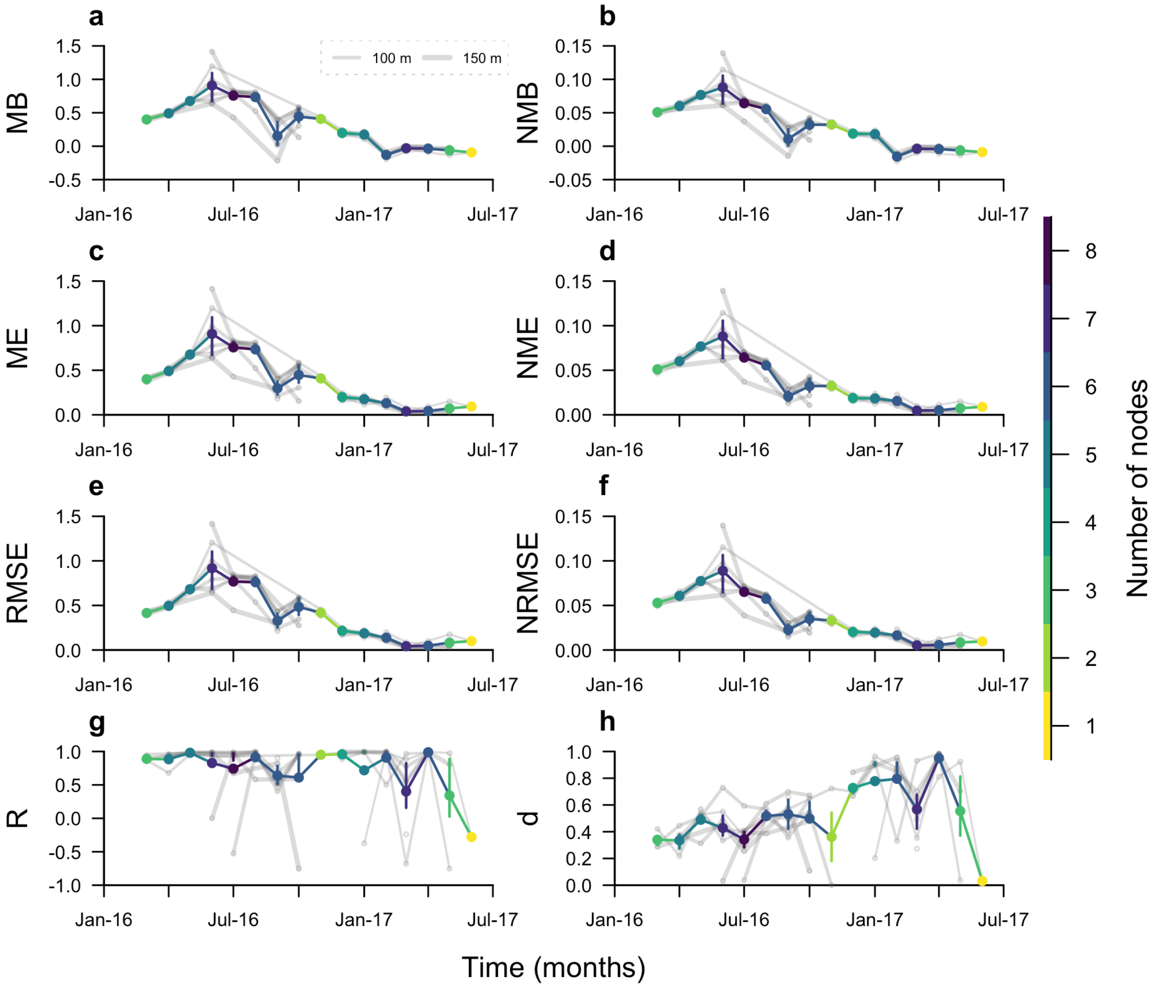

Figure S7. Bottom-temperature model skill metrics by month for nodes in deep (≥ 50 m) areas. Panels follow Figure S4.

Supplementary figures

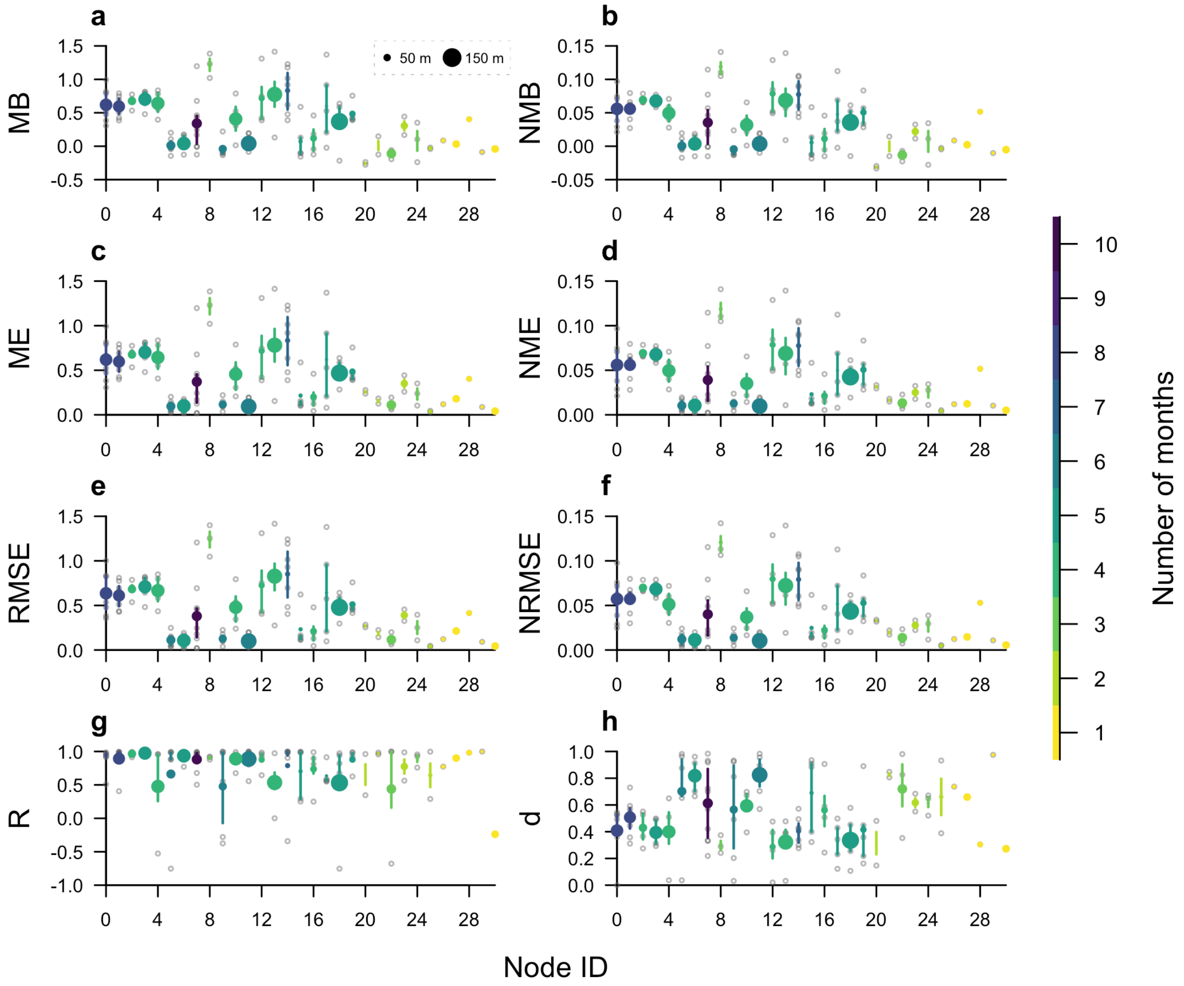

Figure S8. Bottom-temperature model skill metrics by node. Panel metrics follow Figure S4. In each panel, the coloured points indicate the ensemble-average skill score for each node. Points are coloured by the number of analysed months and sized on a continuous scale by depth (see **a**). Vertical error bars show the interquartile range in average skill scores across analysed months for each node. Average skill scores are shown in grey. The points mark monthly values. Node IDs are defined in Table S1.

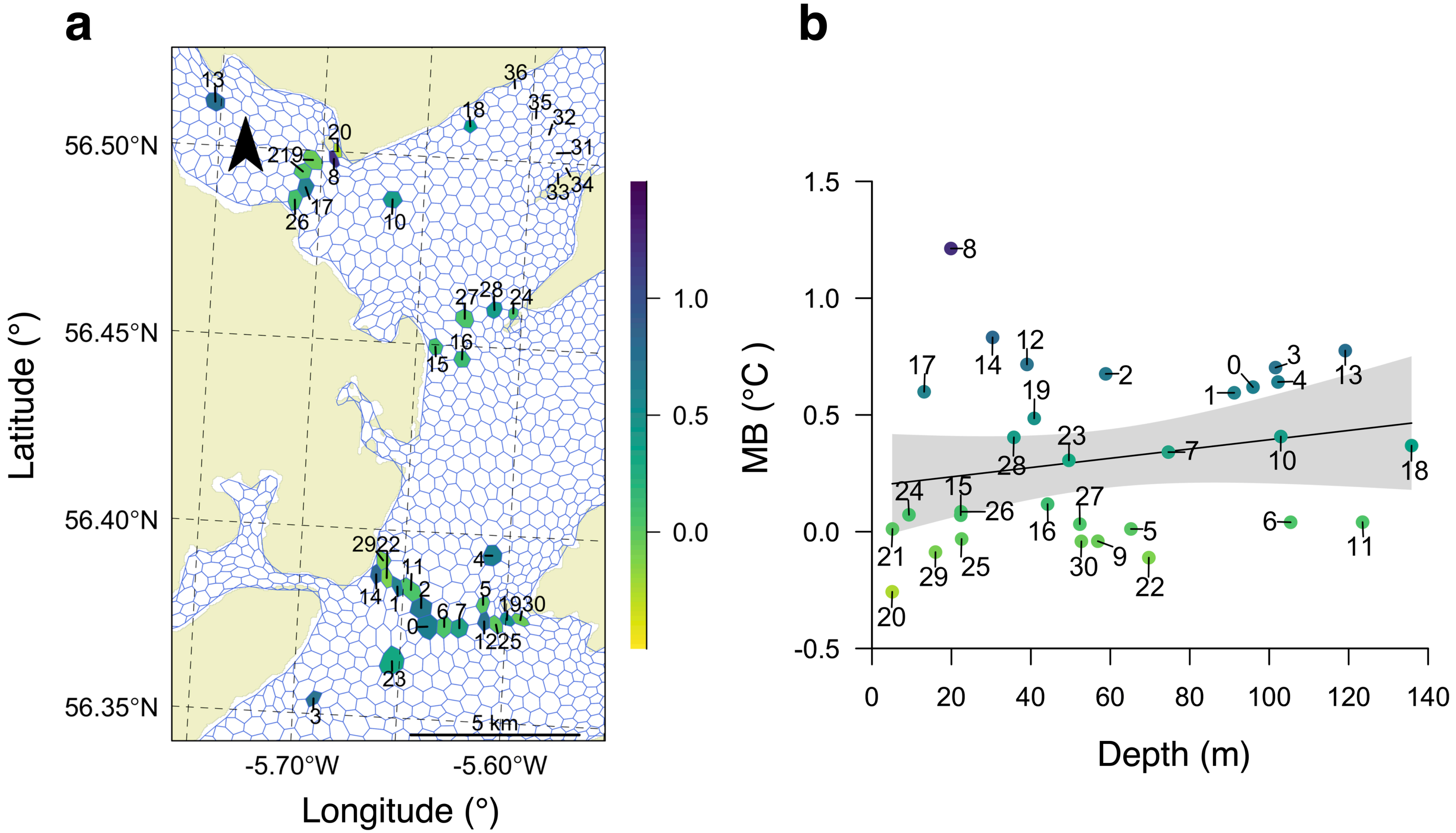

Figure S9. Spatial clustering of ensemble-average Mean Bias (MB) scores. a maps the spatial distribution of MB scores. Map properties follow Figure S1. **b** shows the relationship between ensemble-average MB scores and depth. Points mark nodes and the fitted line and surrounding envelope mark the expected MB and 95 % confidence intervals from a linear model of ensemble-average MB scores in relation to depth. This model only explained 5 % of the variation in ensemble-average MB scores and the effect of depth (estimate = 0.002 ± 0.002 standard error) was statistically insignificant at $p = 0.05$. Note that these analyses excluded the nodes with fewest observations.

Supplementary figures

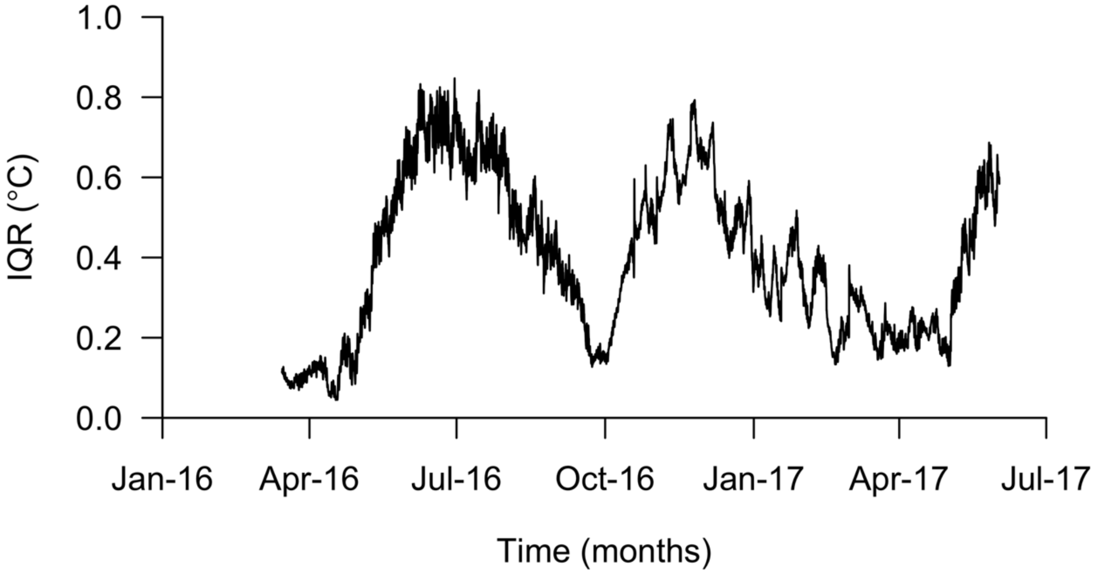

Figure S10. Spatial variation in modelled bottom temperatures across the study site over the study period. Spatial variation is represented as the interquartile range (IQR) in modelled temperatures across all nodes in the Loch Sunart to the Sound of Jura Marine Protected Area for each hour from 15th March 2016 until 1st June 2017.
