# Supplementary material for: Benthic animal-borne sensors and citizen science combine to validate ocean modelling
Source: Sci Rep. 2022 Oct 5;12:16613. doi: 10.1038/s41598-022-20254-z (PMC9534998; doi:10.1038/s41598-022-20254-z)
Supplement: Supplementary file 2 — Supplementary Information 2. [file 41598_2022_20254_MOESM2_ESM.pdf]

**Supplementary information**

# Benthic animal-borne sensors and citizen science combine to validate ocean modelling

**Edward Lavender[1,2*], Dmitry Aleynik[3], Jane Dodd[4], Janine Illian[5], Mark James[2], Sophie Smout[1,2,7], James Thorburn[2,6,7]**

[1]Centre for Research into Ecological and Environmental Modelling, University of St Andrews, St Andrews, United Kingdom

[2]Scottish Oceans Institute, University of St Andrews, St Andrews, United Kingdom

[3]Scottish Association for Marine Science, Oban, United Kingdom

[4]NatureScot, Oban, United Kingdom

[5]School of Mathematics and Statistics, University of Glasgow, Glasgow, United Kingdom

[6]School of Biological Sciences, Queen's University Belfast, Belfast, United Kingdom

*** Correspondence:**

Edward Lavender

el72@st-andrews.ac.uk

[7]These authors jointly supervised this work.

**Supplementary information**

## 1. The West Scotland Coastal Ocean Modelling System

The West Scotland Coastal Ocean Modelling System (WeStCOMS) is a regional ocean model that resolves hourly hydrodynamic conditions for the west coast of Scotland[1,2]. WeStCOMS is nested within the North-East Atlantic Regional Ocean Modelling System[3] at its open boundary. In September 2016, independent temperature observations were obtained for model validation from four sea- and Slocum glider missions near the southwestern boundary of the model domain (between the Isle of Barra and Malin Head). Comparisons between the observed and modelled temperature profiles identified a positive temperature bias (0.3–1.5 °C) in this region from the surface to the seabed. Tests with various corrections applied to the parent temperature profiles showed that the bias was reduced to a minimum by subtracting the average bias value (0.5 °C) from the parent temperature profiles at the open boundary points. In 2017, the external forcing system for the ocean model was updated with this adjustment. However, the effects of this adjustment across the model's domain remained to be fully validated.

## 2. Validation datasets

Temperature observations collected from flapper skate (*Dipturus intermedius*) during (a) undisturbed activity (i.e., bottom temperatures) and (b) individual ascents associated with recreational angling events (i.e., temperature-depth profiles) were identified as sources of validation data for WeStCOMS. The use of these data for validation assumes that they provide an accurate and unbiased measure of depth and/or local water temperature.

According to the manufacturer, Star Oddi Milli-TD archival tags record pressure (depth) instantaneously at pre-programmed intervals to a resolution of 0.24 m and an accuracy of ± 0.6

% of the tag's depth range (5–800 m) or 4.77 m. Temperature sensors record temperature simultaneously to a resolution of 0.032 °C and an accuracy of 0.1 °C. Each tag is individually calibrated between -1 °C to +40 °C in each set point and a calibration certificate is provided. In both cases, depth and temperature measurements are essentially 'snapshot' values (over 120 ms, the sensor takes multiple measurements and returns an average.) However, unlike the instantaneous depth sensors, the temperature sensors have a response time of (up to) 12 s that depends on the temperature gradient alongside other factors. For the analysis of bottom-temperatures (a), these uncertainties were negligible (see Results). For the analysis of ascending temperature-depth profiles (b), the uncertainty in the depth of temperature observations was also limited (by small temperature gradients [< 0.5 °C] and relatively slow ascent rates [median = 0.04 $ms^{-1}$]) relative to the differences between observed and modelled profiles (see Results and Fig. 5).

Temperature-depth profiles collected immediately following tag deployment or recreational angling events that occurred during individuals' time at liberty by descending individuals were also considered as a potential source of validation data, but their utility was limited by the rapid movements of descending individuals, temperature sensor lags (associated with the strong air–water temperature gradient) and uncertainty in individual location. Following release, individuals typically descended rapidly (to depths of 6–126 m at the time of the first sub-surface observation) while temperature sensors continued to record surface values (up to 22.5 °C). This rendered the first sub-surface observation unusable, while the second and third sub-surface observations were not recorded until depths of 64–103 m on average (approximately half to two-thirds of the way down the water column in angling sites). Additionally, unlike ascents, when individuals are attached via rod and line to an angling vessel, individual locations during these descents are less certain. In other species, rapid escape movements away from angling

sites have been observed[4] and it seems likely that flapper skate may exhibit similar behaviours, particularly in areas with strong currents[5]. Given these caveats, temperature-depth profiles from descending individuals were not used for validation in this study.

## 3. Bottom-temperature validation

### 3.1. Dataset assembly

To assemble a dataset for model validation, we used nearest neighbour interpolation to match observed and modelled temperatures. We examined the validity of nearest neighbour interpolation in space by summarising the distribution of absolute differences between the hourly predictions for each node in the validation dataset and neighbouring nodes over the period of observations (15th March 2016 to 1st June 2017). Similarly, we examined the validity of nearest neighbour interpolation in time by summarising the distribution of differences between sequential hourly predictions at each node over the same time period. These analyses showed that small-scale temperature variation (among neighbouring nodes and between sequential hours) was minimal, suggesting that nearest neighbour interpolation was appropriate (see Results).

### 3.2. Model skill metrics

#### 3.2.1. Overall model skill

Overall model skill was quantified using five standard regression metrics:

**Supplementary information**

A. **Pearson's Product Moment Correlation Coefficient ($\boldsymbol{R}$).** $R$ is a measure of the strength and direction of the correlation between modelled ($M$) and observed ($O$) temperatures, defined as

$$R = \frac{\sum_{i=1}^{n}(M_i - \widehat{M})(O_i - \hat{O})}{\sqrt{\sum_{i=1}^{n}(M_i - \widehat{M})^2}\sqrt{\sum_{i=1}^{n}(O_i - \hat{O})^2}} \tag{1}$$

where $\widehat{M}$ and $\hat{O}$ are the mean modelled and observed bottom temperatures and $i$ indexes observations $(1, \dots, n)$.

B. **Index of Agreement ($\boldsymbol{d}$).** $d$ is a measure of the correspondence between predictions and observations that varies from zero (no agreement) to one (perfect agreement)[6], defined as

$$d = 1 - \frac{\sum_{i=1}^{n}(M_i - O_i)^2}{\sum_{i=1}^{n}\left(|M_i - \hat{O}| + |O_i - \hat{O}|\right)^2} \tag{2}$$

C. **Mean Bias ($\boldsymbol{MB}$).** $MB$ is the mean difference between predictions and observations, i.e.,

$$MB = \frac{\sum_{i=1}^{n}(M_i - O_i)}{n} \tag{3}$$

D. **Mean Error ($\boldsymbol{ME}$).** $ME$ is the mean absolute difference between predictions and observations, i.e.,

$$ME = \frac{\sum_{i=1}^{n}|M_i - O_i|}{n} \tag{4}$$

E. **Root Mean Square Error ($\boldsymbol{RMSE}$).** $RMSE$ is the root mean of the squared differences between predictions and observations, i.e.,

$$RMSE = \sqrt{\frac{\sum_{i=1}^{n}(M_i - O_i)^2}{n}} \tag{5}$$

**3.2.2. Spatiotemporal variation in model skill**

**Supplementary information**

We also examined the change in model skill over time and space using model skill metrics. For this analysis, alongside the metrics mentioned above, we considered the normalised $MB$, the normalised $ME$ and the normalised $RMSE$ to account for variation in average temperature. These metrics were defined as follows.

F. **Normalised $\boldsymbol{MB}$ ($\boldsymbol{NMB}$).** $NMB$ is the $MB$ scaled by the mean of the observations:

$$NMB = \frac{MB}{\hat{O}} = \frac{\sum_{i=1}^{n}(M_i - O_i)}{\sum_{i=1}^{n} O_i} \quad (6)$$

where $i$ indexes observations within node/month categories.

G. **Normalised $\boldsymbol{ME}$ ($\boldsymbol{NME}$).** $NME$ is the $ME$ scaled by the mean of the observations:

$$NME = \frac{ME}{\hat{O}} = \frac{\sum_{i=1}^{n}|M_i - O_i|}{\sum_{i=1}^{n} O_i} \quad (7)$$

H. **Normalised $\boldsymbol{RMSE}$ ($\boldsymbol{NRMSE}$).** $NRMSE$ is the $RMSE$ scaled by the mean of the observations:

$$NRMSE = \frac{RMSE}{\hat{O}} \quad (8)$$

## 3.3. Wider patterns of spatiotemporal variation

To contextualise ensemble-average skill scores, we investigated spatiotemporal variation in modelled bottom temperatures across the study site using two analyses.

A. **Temporal variation.** We quantified short-term temporal variation in modelled temperatures as the daily range in modelled bottom temperatures at each node in the study site (specifically the Loch Sunart to the Sound of Jura Marine Protected Area [LStSJ MPA]) over the period of observations.

B. **Spatial variation.** We quantified spatial variation in modelled bottom temperatures as the hourly interquartile range (IQR) in modelled bottom temperatures across the LStSJ MPA over the period of observations.

For each analysis, we visualised the results using a time series plot and took (a) the median daily range or (b) the median hourly IQR as estimates of the average magnitude of variation in bottom temperature in the study site (for the relevant time period).

## 4. Temperature-depth profile validation

### 4.1. Observed profiles

We used temperature-depth profiles collected from ascending tagged individuals during recreational angling events to validate modelled temperature-depth profiles. For this analysis, angling events were identified in a mark-recapture database[5]. Only events for which coordinates were recorded were considered. Potentially erroneous locations were flagged if the maximum observed depth in the recorded location was more than 25 m deeper than the depth of the seabed in that location, given 1 arc-second resolution bathymetry data for the study site from Digimap[7].

### 4.2. Observational uncertainty

Uncertainty in observed temperatures and the depths at which they were sampled was quantified according to manufacturer specifications (see §2), accounting for sensor accuracy, sensor response times and individual ascent rates. Uncertainty in temperature was taken at the manufacturer-quoted value of ± 0.1 °C. Uncertainty in the depths at which temperatures were

sampled was calculated from the accuracy of the depth sensor (± 4.77 m), the temperature sensor response time (12 s) and the rate of ascent between sequential observations as follows.

At any time step ($t$), the true depth of an individual (denoted $A_t$) must lie in the interval $[\alpha_t - 4.77, \alpha_t + 4.77]$, where $\alpha_t$ is the recorded depth, $\alpha_t$ – 4.77 is the shallow-depth limit and $\alpha_t + 4.77$ is the deep-depth limit. Thus, for any pair of time steps (say, $t = 1$ and $t = 2$), the individual's true depth must lie between $[\alpha_{t=1} - 4.77, \alpha_{t=1} + 4.77]$ and $[\alpha_{t=2} - 4.77, \alpha_{t=2} + 4.77]$ respectively. If we define $A_{t=1}$ as the individual's true depth at the start of an angling event, we can assume that the depth to which the simultaneous temperature observation corresponds (denoted $B_{t=1}$) also lies within the interval $[\alpha_{t=1} - 4.77, \alpha_{t=1} + 4.77]$. This assumption aligns with the way that hooked individuals typically 'dig' into the sediment for a period of time (usually up to ten minutes) before the tension from the angling line forces them into ascent[5].

At $t = 2$, the deep-depth limit for the temperature observation ($B_{t=2}$) is increased (deepened), relative to $\alpha_{t=2} + 4.77$, by the potential lag in the temperature sensor (which is up to 12 s) according to the individual's ascent rate. If the individual moved from $A_{t=1}$ to $A_{t=2} = \alpha_{t=2} + 4.77$ (the deepest possible depth of the individual at $t = 2$), then $B_{t=2}$ may be as deep as

$$(\alpha_{t=2} + 4.77) + \left(\left(A_{t=1} - (\alpha_{t=2} + 4.77)\right)/120\right) \times 12, \quad (9)$$

assuming a constant ascent rate (and given the two-minute resolution of observations). In reality $A_{t=1}$and $A_{t=2}$ are unknown and the maximum possible depth of $B_{t=2}$ depends on the values within $[\alpha_{t=1} - 4.77, \alpha_{t=1} + 4.77]$ and $[\alpha_{t=2} - 4.77, \alpha_{t=2} + 4.77]$ that maximise this equation (9). However, in practice we note that $B_{t=2}$ is likely to be shallower that this maximum limit given the small temperature gradients ascending individuals passed through in this study (see §2).

In terms of the shallow limit for $B_{t=2}$, we have already recognised that the shallowest possible depth of the individual at $t = 2$ is $\alpha_{t=2} - 4.77$. As explained above, if the temperature sensor is delayed (by up to 12 s), the shallowest value for $B_{t=2}$ could be deeper than $\alpha_{t=2} - 4.77$. However, since the sensor has the potential to respond more quickly (essentially instantaneously), we can regard the shallowest value for $B_{t=2}$ as equal to $\alpha_{t=2} - 4.77$.

Using this method, for each profile and temperature observation, we calculated the possible limits for $B_t$. We then visualised temperature-depth observations together with their uncertainties in relation to modelled profiles to examine model skill (see §4.3 and Fig. 5).

## 4.3. Modelled profiles

Observed temperature depth-profiles were compared against modelled profiles for the nearest node and hour, accounting for the tidal elevation at the start of each angling event. We determined that it was not necessary to account for continued change in model layer depth during time on hook (resulting from continued change in tidal elevation) by deriving the distribution of absolute differences in tidal elevation between sequential hours on the day of each angling event. Differences were negligible (see Results).

**Supplementary information**

**References**


1. Aleynik, D., Dale, A. C., Porter, M. & Davidson, K. A high resolution hydrodynamic model system suitable for novel harmful algal bloom modelling in areas of complex coastline and topography. *Harmful Algae* **53**, 102–117 (2016).
2. Davidson, K. *et al.* HABreports: Online early warning of harmful algal and biotoxin risk for the Scottish shellfish and finfish aquaculture industries. *Front. Mar. Sci.* **8**, 631732 (2021).
3. Dabrowski, T., Lyons, K., Berry, A., Cusack, C. & Nolan, G. D. An operational biogeochemical model of the North-East Atlantic: model description and skill assessment. *J. Mar. Syst.* **129**, 350–367 (2014).
4. Hoolihan, J. P. *et al.* Evaluating post-release behaviour modification in large pelagic fish deployed with pop-up satellite archival tags. *ICES J. Mar. Sci.* **68**, 880–889 (2011).
5. Lavender, E. *et al.* Behavioural responses of a large, benthic elasmobranch to catch-and-release angling. *Front. Mar. Sci.* **9**, 864344 (2022).
6. Willmott, C. J. Some comments on the evaluation of model performance. *Bull. Am. Meteorol. Soc.* **63**, 1309–1313 (1982).
7. EDINA Marine Digimap Service. Marine Themes Digital Elevation Model 1 Arc Second [ASC geospatial data], Scale 1:50000. https://digimap.edina.ac.uk (2016).
