# Supplementary material for: Benthic animal-borne sensors and citizen science combine to validate ocean modelling
Source: Sci Rep. 2022 Oct 5;12:16613. doi: 10.1038/s41598-022-20254-z (PMC9534998; doi:10.1038/s41598-022-20254-z)
Supplement: Supplementary file 3 — Supplementary Information 3. [file 41598_2022_20254_MOESM3_ESM.pdf]

Supplementary tables

Benthic animal-borne sensors and citizen science combine to validate ocean modelling

Edward Lavender[1,2*], Dmitry Aleynik[3], Jane Dodd[4], Janine Illian[5], Mark James[2], Sophie Smout[1,2,7], James Thorburn[2,6,7]

[1]Centre for Research into Ecological and Environmental Modelling, University of St Andrews, St Andrews, United Kingdom

[2]Scottish Oceans Institute, University of St Andrews, St Andrews, United Kingdom

[3]Scottish Association for Marine Science, Oban, United Kingdom

[4]NatureScot, Oban, United Kingdom

[5]School of Mathematics and Statistics, University of Glasgow, Glasgow, United Kingdom

[6]School of Biological Sciences, Queen's University Belfast, Belfast, United Kingdom

*** Correspondence:**

Edward Lavender

el72@st-andrews.ac.uk

[7]These authors jointly supervised this work.

Supplementary tables

Table S1. Bottom-temperature validation effort summary. For each node with observations, the identifier (0,…,36), name, number of observations, percentage of total observations and seabed depth (as represented by the model) are shown. Sorted by Node ID (n).

Node ID	Name	n	Percentage (%)	Depth (m)
0	22420	1511	28.73	95.92
1	22423	774	14.71	91.20
2	22421	517	9.83	58.85
3	19887	259	4.92	101.60
4	24203	239	4.54	102.21
5	23837	231	4.39	65.19
6	22778	224	4.26	105.39
7	23139	198	3.76	74.62
8	32046	139	2.64	19.87
9	32048	127	2.41	56.86
10	30410	105	2.00	102.93
11	22781	95	1.81	123.51
12	23836	92	1.75	39.02
13	34662	89	1.69	119.14
14	22784	77	1.46	30.36
15	27988	76	1.44	22.35
16	27463	69	1.31	44.21
17	32384	61	1.16	13.14
18	32038	48	0.91	135.84
19	24577	44	0.84	40.84
20	32377	44	0.84	5.10
21	32383	42	0.80	5.16
22	22424	37	0.70	69.70
23	21355	35	0.67	49.58
24	28813	28	0.53	9.32
25	24198	24	0.46	22.57
26	32385	23	0.44	22.41
27	28257	15	0.29	52.33
28	28522	8	0.15	35.73
29	22783	6	0.11	15.99
30	24576	6	0.11	52.71
31	32366	6	0.11	104.68
32	32704	4	0.08	123.11
33	32364	2	0.04	63.20
34	32700	2	0.04	55.50
35	32706	2	0.04	139.07
36	33074	1	0.02	113.41

Supplementary tables

Table S2. Correlations between monthly bottom-temperature statistics for (a) the relationship between temperature variability, the number of observations and average temperature and (b) model error, the number of observations and average temperature. For each analysis, the correlation functions are shown along with the correlation coefficients. Correlations were calculated from summarises for each month of the time series. M and O indicate modelled and observed temperatures; $\widehat{M}$, $\widehat{O}$ and $\widehat{\Delta T}$ indicate the mean modelled temperature, observed temperature and temperature difference across all observations (i) in each month, where $\Delta T_i = M_i - O_i$; σ indicates standard deviation; and n indicates sample size. There are weak–moderate positive correlations between temperature variability, the number of observations and the average temperature (for both modelled and observed temperatures) and between model error, the number of observations and the average temperature. These results led to a simulation-based analysis of model skill metrics designed to control for these correlations.

Question	Correlation	*R*
**a.** Is there an increase in bottom temperature variability with the number of observations or the average temperature?	$cor(\sigma_O, n)$	0.25
	$cor(\sigma_M, n)$	0.26
	$cor(\sigma_O, \widehat{O})$	0.55
	$cor(\sigma_M, \widehat{M})$	0.37
**b.** Is there an increase in model error (the difference between modelled and observed bottom temperatures) with the number of observations or the average temperature?	$cor(\widehat{\Delta T}, n)$	0.66
	$cor(\widehat{\Delta T}, \widehat{O})$	0.38
	$cor(\widehat{\Delta T}, \widehat{M})$	0.51

Supplementary tables

Table S3. Ensemble-average skill scores for bottom temperature by month. For each month (from March 2016 until June 2017), the number of nodes with observations available for validation (n_{node}), the ensemble-average mean modelled (M) and observed (O) temperature ($\widehat{M}$ and $\widehat{O}$), the ensemble-average standard deviations (σ_M and σ_O) and ensemble-average skill scores for the following metrics are shown: Mean Bias (MB), Normalised Mean Bias (NMB), Mean Error (ME), Normalised Mean Error (NME), Root Mean Square Error ($RMSE$), Normalised Root Mean Square Error ($NRMSE$), Correlation Coefficient (R) and Index of Agreement (d). Note the absence of a value for R for November 2016: in this month, only one node recorded a sufficient number of observations for inclusion in the analysis; all observations occurred within a period of five hours on 2016-11-01 and were identical (13.32 °C), precluding estimation of R. Units are °C except for n_{node}, NMB, NME, $NRMSE$, R and d which are unitless. Sorted by time.

Time (months)	n_{node}	$\widehat{M}$	$\widehat{O}$	σ_M	σ_O	MB	NMB	ME	NME	$RMSE$	$NRMSE$	R	d
2016-03	7	8.24	7.85	0.04	0.15	0.40	0.05	0.40	0.05	0.41	0.05	0.93	0.36
2016-04	8	8.62	8.14	0.13	0.11	0.48	0.06	0.48	0.06	0.48	0.06	0.89	0.30
2016-05	9	9.65	8.89	0.37	0.29	0.76	0.09	0.76	0.09	0.77	0.09	0.98	0.46
2016-06	10	11.37	10.36	0.27	0.29	1.01	0.10	1.01	0.10	1.01	0.10	0.83	0.36
2016-07	11	12.72	11.82	0.35	0.26	0.90	0.08	0.90	0.08	0.91	0.08	0.80	0.35
2016-08	8	14.07	13.26	0.23	0.39	0.81	0.06	0.81	0.06	0.84	0.06	0.88	0.48
2016-09	9	14.66	14.41	0.12	0.28	0.25	0.02	0.36	0.02	0.40	0.03	0.51	0.50
2016-10	8	14.32	13.88	0.27	0.36	0.44	0.03	0.44	0.03	0.47	0.03	0.70	0.57
2016-11	3	13.04	12.63	0.22	0.14	0.41	0.03	0.41	0.03	0.44	0.03	-	0.31
2016-12	7	10.67	10.37	0.18	0.21	0.31	0.03	0.31	0.03	0.34	0.03	0.73	0.59
2017-01	9	9.66	9.49	0.25	0.23	0.17	0.02	0.17	0.02	0.18	0.02	0.79	0.71
2017-02	10	8.26	8.43	0.18	0.19	-0.16	-0.02	0.16	0.02	0.17	0.02	0.91	0.73
2017-03	11	7.97	8.03	0.03	0.03	-0.06	-0.01	0.06	0.01	0.07	0.01	0.42	0.50
2017-04	10	8.37	8.43	0.15	0.17	-0.06	-0.01	0.06	0.01	0.07	0.01	0.99	0.93
2017-05	6	9.71	9.72	0.17	0.13	0.00	0.00	0.10	0.01	0.12	0.01	0.56	0.62
2017-06	1	10.36	10.45	0.04	0.00	-0.09	-0.01	0.09	0.01	0.10	0.01	-0.28	0.03

Supplementary tables

Table S4. Improvement in ensemble-average skill scores for bottom temperature between 2016 and 2017. For each metric (Mean Bias, MB; Mean Error, ME; Root Mean Square Error, $RMSE$; Correlation Coefficient, R; Index of Agreement, d), for each overlapping month in 2016–17 (March–May), the number of nodes with observations (n_{node}), the ensemble-average skill score ($score$), the improvement (I) and percentage improvement (P) in the ensemble-average skill score are shown. Improvements are calculated as $I = (score_{2016} - score_{2017})\emptyset$ where $score$ is the ensemble-average skill score and $\emptyset$ is a variable that takes a value of -1 for MB, ME and $RMSE$ and a value of 1 otherwise (ensuring that positive values for all metrics imply improvement). Metric units are °C except for R and d which are unitless.

Metric	Period	$n_{node,2016}$	$n_{node,2017}$	$score_{2016}$	$score_{2017}$	I	P (%)
MB	March	7	11	0.40	-0.06	0.45	114.06
	April	8	10	0.48	-0.06	0.53	112.08
	May	9	6	0.76	0.00	0.75	99.44
	**Mean**	**8**	**9**	**0.54**	**-0.04**	**0.58**	**108.53**
ME	March	7	11	0.40	0.06	0.34	84.50
	April	8	10	0.48	0.06	0.42	87.02
	May	9	6	0.76	0.10	0.66	86.87
	**Mean**	**8**	**9**	**0.54**	**0.07**	**0.47**	**86.13**
$RMSE$	March	7	11	0.41	0.07	0.34	83.59
	April	8	10	0.48	0.07	0.41	85.77
	May	9	6	0.77	0.12	0.65	84.94
	**Mean**	**8**	**9**	**0.55**	**0.08**	**0.47**	**84.77**
R	March	7	11	0.93	0.42	-0.51	-54.84
	April	8	10	0.89	0.99	0.10	11.30
	May	9	6	0.98	0.56	-0.42	-43.00
	**Mean**	**8**	**9**	**0.93**	**0.66**	**-0.28**	**-28.85**
d	March	7	11	0.36	0.50	0.14	40.02
	April	8	10	0.30	0.93	0.63	210.00
	May	9	6	0.46	0.62	0.16	34.35
	**Mean**	**8**	**9**	**0.37**	**0.68**	**0.31**	**94.79**

Supplementary tables

Table S5. Correlations between node-wise bottom-temperature statistics for (a) the relationship between temperature variability, the number of observations and average temperature and (b) model error, the number of observations and average temperature. Table properties follow Table S2.

Question	Correlation	*R*
**a.** Is there an increase in bottom temperature variability with the number of observations or the average temperature?	$cor(\sigma_o, n)$	0.45
	$cor(\sigma_M, n)$	0.43
	$cor(\sigma_o, \hat{O})$	0.28
	$cor(\sigma_M, \hat{M})$	0.24
**b.** Is there an increase in model error (the difference between modelled and observed bottom temperatures) with the number of observations or the average temperature?	$cor(\widehat{\Delta T}, n)$	0.36
	$cor(\widehat{\Delta T}, \hat{O})$	0.40
	$cor(\widehat{\Delta T}, \hat{M})$	0.53

Supplementary tables

Table S6. Model skill metrics for bottom temperature by node. Column definitions follow Table S3, with the exception of n_{time} which defines the number of months with observations for each node. Note that only nodes with sufficient data (0–30) were included in the analysis. Sorted by Node ID.

ID	n_{time}	$\widehat{M}$	$\widehat{O}$	σ_M	σ_O	ME	NMB	ME	NME	$RMSE$	$NRMSE$	R	d
0	8	12.34	11.72	0.23	0.26	0.62	0.06	0.62	0.06	0.64	0.06	0.89	0.41
1	8	11.70	11.10	0.30	0.34	0.59	0.06	0.60	0.06	0.61	0.06	0.89	0.51
2	4	10.47	9.79	0.30	0.27	0.68	0.07	0.68	0.07	0.68	0.07	0.97	0.43
3	5	11.11	10.41	0.20	0.24	0.70	0.07	0.70	0.07	0.71	0.07	0.97	0.39
4	4	13.94	13.30	0.21	0.27	0.64	0.05	0.64	0.05	0.67	0.05	0.48	0.40
5	6	8.96	8.95	0.18	0.19	0.01	0.00	0.10	0.01	0.11	0.01	0.66	0.70
6	5	9.02	8.98	0.15	0.14	0.04	0.00	0.10	0.01	0.11	0.01	0.94	0.82
7	10	9.58	9.24	0.20	0.19	0.34	0.04	0.37	0.04	0.38	0.04	0.88	0.61
8	3	11.52	10.30	0.40	0.29	1.21	0.12	1.21	0.12	1.23	0.12	0.90	0.30
9	6	9.05	9.09	0.14	0.12	-0.04	0.00	0.12	0.01	0.13	0.01	0.48	0.57
10	4	13.91	13.51	0.20	0.32	0.41	0.03	0.46	0.04	0.48	0.04	0.89	0.59
11	6	9.11	9.06	0.17	0.18	0.04	0.00	0.09	0.01	0.10	0.01	0.89	0.82
12	4	9.46	8.75	0.14	0.14	0.72	0.08	0.72	0.08	0.72	0.08	0.88	0.29
13	4	13.12	12.35	0.13	0.22	0.78	0.07	0.78	0.07	0.83	0.07	0.54	0.32
14	7	11.55	10.72	0.28	0.32	0.83	0.08	0.83	0.08	0.85	0.08	0.79	0.40
15	5	9.00	8.94	0.19	0.20	0.07	0.01	0.22	0.02	0.23	0.02	0.70	0.69
16	4	9.05	8.93	0.13	0.13	0.12	0.01	0.20	0.02	0.21	0.02	0.74	0.56
17	5	12.38	11.78	0.15	0.19	0.60	0.05	0.62	0.05	0.64	0.05	0.64	0.32
18	5	11.99	11.63	0.14	0.16	0.37	0.04	0.47	0.04	0.48	0.04	0.53	0.34
19	5	10.76	10.27	0.26	0.26	0.49	0.05	0.49	0.05	0.51	0.05	0.88	0.42
20	2	7.92	8.19	0.10	0.11	-0.26	-0.03	0.26	0.03	0.27	0.03	0.65	0.31
21	2	9.31	9.31	0.25	0.21	0.01	0.00	0.15	0.02	0.18	0.02	0.97	0.83
22	3	8.21	8.33	0.15	0.18	-0.11	-0.01	0.11	0.01	0.12	0.01	0.44	0.72
23	2	14.45	14.15	0.27	0.35	0.31	0.02	0.35	0.03	0.39	0.03	0.77	0.62
24	3	9.43	9.36	0.18	0.17	0.07	0.01	0.23	0.02	0.25	0.03	0.88	0.61
25	2	8.35	8.38	0.06	0.06	-0.03	0.00	0.04	0.00	0.04	0.00	0.64	0.66

Supplementary tables

ID	n_{time}	$\widehat{M}$	$\widehat{O}$	σ_M	σ_O	ME	NMB	ME	NME	$RMSE$	$NRMSE$	R	d
26	1	10.07	10.01	0.13	0.11	0.09	0.01	0.12	0.01	0.12	0.01	0.77	0.74
27	1	14.61	14.57	0.09	0.31	0.03	0.00	0.18	0.01	0.21	0.01	0.90	0.66
28	1	8.24	7.83	0.02	0.12	0.40	0.05	0.40	0.05	0.41	0.05	0.98	0.30
29	1	8.37	8.46	0.33	0.31	-0.09	-0.01	0.09	0.01	0.09	0.01	1.00	0.97
30	1	7.99	8.03	0.01	0.01	-0.04	-0.01	0.04	0.01	0.04	0.01	-0.24	0.27

Supplementary tables

Table S7. A summary of capture events. For each retrieved archival tag, the capture event type (a recreational angling event that occurred during [D] the individual's time at liberty or the final [F] tag retrieval event), the site (Kerrera [K] or Loch Sween [LS]), date, start depth and temperature, as recorded by the archival tag, are shown. Of the nine events for which capture locations were recorded, eight contributed temperature-depth observations used for validation. (The depth for the capture event recorded in Loch Sween* is inconsistent with the depth time series around capture for that individual given that the maximum depth for the area reported by the Loch Sween Possible Nature Conservation Marine Protected Area Data Confidence Assessment[1] is 40 m.) Site coordinates are not shown because anglers' favoured fishing marks are sensitive.

Tag ID	Event	Site	Date	Depth (m)	T (°C)
1502	F	-	2019-02-26	155.11	8.28
1507	F	-	2016-05-18	166.52	9.01
1509	F	K	2017-04-20	150.87	8.52
1511	F	K	2016-08-28	146.00	13.68
1512	F	-	2016-07-13	162.62	11.82
1518	F	K	2017-03-22	96.69	8.02
1519	F	-	2019-03-31	151.08	8.12
1520	F	-	2016-07-13	138.14	11.84
1522	D	-	2016-07-13	140.39	11.84
1522	D	K	2016-08-27	143.14	13.63
1522	F	K	2017-04-18	144.58	8.49
1523	D	-	2016-05-16	157.04	8.96
1523	F	-	2016-05-26	158.23	9.46
1525	F	-	2017-05-03	139.59	8.94
1526	F	-	2016-03-19	184.00	7.75
1533	D	-	2016-05-10	172.63	8.70
1533	F	K	2016-10-08	163.36	14.02
1536	D	LS*	2016-04-29	134.17	8.26
1536	F	K	2016-10-23	153.21	13.60
1538	F	-	2017-07-19	83.90	12.11
1539	F	-	2016-04-29	136.01	8.27
1547	F	-	2018-04-26	147.33	7.14
1548	F	K	2017-04-21	137.20	8.51
1552	F	-	2016-04-02	149.18	7.86
1558	F	-	2016-05-28	145.03	9.51
1574	F	-	2016-06-09	167.80	10.35

[1]Scottish Natural Heritage. *Scottish MPA project data confidence assessment: Loch Sween Possible Nature Conservation MPA*. https://apps.snh.gov.uk/sitelink-api/v1/sites/10419/documents/50 (2013).
